# Capacitance measurement of dendritic exocytosis in an electrically coupled inhibitory retinal interneuron: an experimental and computational study

**DOI:** 10.14814/phy2.14186

**Published:** 2019-08-04

**Authors:** Espen Hartveit, Margaret Lin Veruki, Bas‐Jan Zandt

**Affiliations:** ^1^ Department of Biomedicine University of Bergen Bergen Norway

**Keywords:** AII amacrine cell, capacitance, compartmental model, exocytosis, glycine, inhibitory interneuron, presynaptic, retina

## Abstract

Exocytotic release of neurotransmitter can be quantified by electrophysiological recording from postsynaptic neurons. Alternatively, fusion of synaptic vesicles with the cell membrane can be measured as increased capacitance by recording directly from a presynaptic neuron. The “Sine + DC” technique is based on recording from an unbranched cell, represented by an electrically equivalent RC‐circuit. It is challenging to extend such measurements to branching neurons where exocytosis occurs at a distance from a somatic recording electrode. The AII amacrine is an important inhibitory interneuron of the mammalian retina and there is evidence that exocytosis at presynaptic lobular dendrites increases the capacitance. Here, we combined electrophysiological recording and computer simulations with realistic compartmental models to explore capacitance measurements of rat AII amacrine cells. First, we verified the ability of the “Sine + DC” technique to detect depolarization‐evoked exocytosis in physiological recordings. Next, we used compartmental modeling to demonstrate that capacitance measurements can detect increased membrane surface area at lobular dendrites. However, the accuracy declines for lobular dendrites located further from the soma due to frequency‐dependent signal attenuation. For sine wave frequencies ≥1 kHz, the magnitude of the total releasable pool of synaptic vesicles will be significantly underestimated. Reducing the sine wave frequency increases overall accuracy, but when the frequency is sufficiently low that exocytosis can be detected with high accuracy from all lobular dendrites (~100 Hz), strong electrical coupling between AII amacrines compromises the measurements. These results need to be taken into account in studies with capacitance measurements from these and other electrically coupled neurons.

## Introduction

During chemical synaptic transmission, neurotransmitter is released by exocytosis of synaptic vesicles from the presynaptic neuron and, after diffusion across the synaptic cleft, it can bind to ionotropic and metabotropic receptors on the postsynaptic neuron (reviewed by Südhof and Rizo 2012; Helmchen and Nägerl 2016; Silver et al. 2016). This process can be studied quantitatively by measuring the magnitude and time course of the postsynaptic current via electrophysiological whole‐cell recording. It is also of interest, however, to measure neurotransmitter release directly from the presynaptic side, without the interference of postsynaptic mechanisms. Capacitance measurement of exocytosis is based on measuring the increase of surface area after the vesicular membrane has fused with the plasma membrane (Lindau and Neher 1988; Gillis 1995). When standard capacitance measurement techniques are applied to an unbranched, round cell using whole‐cell recording and sine wave voltage commands, the cell is represented by a simple, electrically equivalent RC‐circuit. Because neurons are branched structures with varying degrees of complex geometry, there has been considerable interest in the possibility of extending capacitance measurements from cells with simple and compact geometry to more general classes of neurons with complex branching (Kushmerick and von Gersdorff 2003; Kim and von Gersdorff 2010). This includes whole‐cell recordings from mossy fiber boutons in the hippocampus (Hallermann et al. 2003), axon terminals of goldfish Mb1 bipolar cells (Heidelberger et al. 1994; von Gersdorff and Matthews 1999) and rat rod bipolar cells (Oltedal and Hartveit 2010), axon terminals of the brainstem calyx of Held (Sun and Wu 2001; Wölfel and Schneggenburger 2003), and axon terminals of neurons in the posterior pituitary gland (Hsu and Jackson 1996). For these cases, whole‐cell patch‐clamp recordings were performed with the pipette located at, or in the immediate vicinity of, the subcellular compartment where exocytosis is considered to take place. In addition, attempts have been made to use whole‐cell recordings from the cell bodies of small neurons to measure exocytosis occurring at varying locations along neuronal processes, including soma‐end recordings from isolated mouse rod bipolar cells (Zhou et al. 2006). Ideally, when capacitance measurements are attempted from morphological structures with essentially arbitrary geometry that cannot be represented as simple RC‐circuits, the conditions for optimal detection of exocytosis should be explored with the help of compartmental models developed from physiological recording and quantitative morphological reconstruction (e.g. Hallermann et al. 2003; Oltedal and Hartveit 2010).

Recently, capacitance measurements of exocytosis using somatic whole‐cell recordings were extended to AII amacrine cells in mouse retina (Balakrishnan et al. 2015). Compared to retinal bipolar cells, AII amacrines have a considerably more complex geometry, with extensively branching dendritic trees (Zandt et al. 2017). These cells receive excitatory glutamatergic input from rod bipolar cells at their arboreal dendrites in the proximal part of the inner plexiform layer (Kolb 1979; Sterling et al. 1988; Strettoi et al. 1990, 1992; Singer and Diamond 2003; Veruki et al. 2003). At these dendrites they are also connected via gap junctions (functioning as electrical synapses), both to other AII amacrine cells (Kolb and Famiglietti 1974; Sterling 1983; Strettoi et al. 1992; Chun et al. 1993; Veruki and Hartveit 2002a) and to ON‐cone bipolar cells (Kolb and Famiglietti 1974; Strettoi et al. 1992, 1994; Tsukamoto et al. 2001; Veruki and Hartveit 2002b). In the distal part of the inner plexiform layer, AII amacrine cells receive excitatory glutamatergic input from OFF‐cone bipolar cells at their lobular dendrites and appendages (McGuire et al. 1984; Strettoi et al. 1992, 1994; Tsukamoto et al. 2001; Veruki et al. 2003; Graydon et al. 2018). The lobular appendages are also presynaptic to OFF‐cone bipolar cells and OFF‐ganglion cells at inhibitory glycinergic synapses (McGuire et al. 1984; Pourcho and Goebel 1985; Strettoi et al. 1992, 1994; Sassoè‐Pognetto et al. 1994; Tsukamoto et al. 2001; Graydon et al. 2018). By measuring the increase of capacitance evoked by activation of voltage‐gated Ca^2+^ channels under different conditions, Balakrishnan et al. (2015) were able to characterize a series of functionally important properties of the glycinergic synapses of AII amacrine cells. However, capacitance measurements from AII amacrine cells have not yet been validated or explored with computer simulations using realistic compartmental models. Here, we take advantage of the recent development of such models (Zandt et al. 2018) to study the optimal conditions for capacitance measurements of AII amacrine cells, including the potential to measure increased capacitance following exocytosis at the lobular appendages. The main conclusion from our study is that capacitance measurement of dendritic exocytosis is indeed possible for these geometrically complex neurons. However, the electrotonic attenuation from the soma during a whole‐cell recording is sufficiently strong that standard capacitance measurements will not be able to detect the total releaseable pools of vesicles. If the measurement parameters are modified to compensate for this, the electrical coupling of AII amacrine cells will compromise the results. Future studies with capacitance measurements of exocytosis from these and similarly complex neurons with electrical coupling will need to take these results into account.

## Methods

### Retinal slice preparation

General aspects of the methods have previously been described in detail (Hartveit, 1996). The use of animals in this study was carried out under the approval of and in accordance with the regulations of the Animal Laboratory Facility at the Faculty of Medicine at the University of Bergen (accredited by AAALAC International). Albino rats (4–7 weeks postnatal, female) had ad libitum access to food and water and were kept on a 12/12 light/dark cycle. Animals were deeply anesthetized with isoflurane in oxygen and killed by cervical dislocation. After removing the eyes and dissecting out the retinas, retinal slices were cut by hand with a curved scalpel blade at a thickness of ~100 to ~150 *µ*m. The slices were visualized using an upright microscope (BX51WI; Olympus or Axioskop FS; Zeiss) with a ×60 (0.9 NA; Olympus) or ×40 (0.75 NA; Zeiss) water immersion objective and infrared differential interference contrast (IR‐DIC) videomicroscopy. Recordings were carried out at room temperature (22–25°C).

### Solutions and drugs

The extracellular perfusing solution was continuously bubbled with 95% O_2_–5% CO_2_ and had the following composition (in mmol/L): 125 NaCl, 25 NaHCO_3_, 2.5 KCl, 2.5 CaCl_2_, 1 MgCl_2_, 10 glucose, pH 7.4 (osmolarity ~300 mOsm). For capacitance measurements, the recording pipettes were filled with (in mmol/L): 80 Cs‐methanesulfonate (CsCH_3_SO_3_), 40 CsCl, 10 tetraethylammonium chloride (TEA‐Cl), 28 Hepes, 2 EGTA, 3 MgATP, 1 Na_3_GTP (pH adjusted to 7.3 with CsOH). For visualization of the cells using fluorescence microscopy after the recording, the pipette solution contained Alexa Fluor 594 hydrazide (Alexa 594) as sodium salt (40 *µ*mol/L; Invitrogen/Thermo Fisher Scientific). In experiments with simultaneous dual recording from pairs of synaptically coupled cells, the pipettes were filled with one of two different solutions (to obtain different chloride equilibrium potentials; *E*_Cl_). The first (“low chloride”; *E*_Cl_ ~−90 mV) contained (in mmol/L): 140 K‐gluconate, 5 Hepes, 1 CaCl_2_, 1 MgCl_2_, 5 EGTA, 4 Na_2_ATP (pH adjusted to 7.3 with KOH). The second (“high chloride”; *E*_Cl_ ~ 0 mV) contained (in mmol/L): 125 CsCl, 4 NaCl, 5 Hepes, 1 CaCl_2_, 1 MgCl_2_, 5 EGTA, 15 TEA‐Cl, 4 Na_2_ATP (pH adjusted to 7.3 with CsOH). For visualization of the cells using fluorescence microscopy after the recording, both these pipette solutions contained Lucifer yellow (1 mg/mL). For MPE microscopy and Ca^2+^ imaging, the recording pipettes were filled with (in mmol/L): 83 CsCH_3_SO_3_, 40 CsCl, 10 TEA‐Cl, 28 Hepes, 3 MgATP, 1 Na_3_GTP, 0.2 Oregon Green 488 BAPTA‐1 (OGB‐1; Invitrogen/Thermo Fisher Scientific), and 0.06 Alexa 594.

The theoretical liquid junction potential (the potential of the extracellular solution relative to that of the intracellular solution) was calculated with the software program JPCalcW (Molecular Devices, Sunnyvale, CA) and all membrane holding potentials (*V*_hold_) were automatically corrected for the liquid junction potential on‐line by the data acquisition software (Pulse or Patchmaster; HEKA Elektronik, Lambrecht/Pfalz, Germany).

Drugs were added directly to the extracellular solution used to perfuse the slices. The concentrations of drugs were as follows (*µ*mol/L; supplier Tocris Bioscience, Bristol, UK; unless otherwise noted): 10 bicuculline methchloride, 1 strychnine (Research Biochemicals Inc., Natick, MA), 10 6‐cyano‐7‐nitroquinoxaline‐2,3‐dione (CNQX), and 20 (RS)‐3‐(2‐carboxypiperazin‐4‐yl)‐propyl‐1‐phosphonic acid (CPP). To block electrical coupling via gap junctions, we added 100 *µ*mol/L 2‐[(2,6‐dichloro‐3‐methylphenyl)amino]benzoic acid sodium salt (meclofenamic acid [MFA] sodium salt; Sigma‐Aldrich) to the extracellular solution (Veruki and Hartveit, 2009). Solutions were either made up freshly for each experiment or were prepared from concentrated aliquots stored at −20°C.

### Electrophysiological recording and data acquisition

Patch pipettes were pulled from thick‐walled borosilicate glass (outer diameter, 1.5 mm; inner diameter, 0.86 mm; Sutter Instrument, Novato, CA). For capacitance measurements, the pipettes were coated with Para‐film (American National Can; Greenwich, CT) to reduce their effective capacitance. In addition, the fluid level both in the recording chamber and in the pipette was kept low to minimize the electrode capacitance. The open‐tip resistance of the pipettes ranged from ~5 to ~8 MΩ when filled with intracellular solution. For capacitance measurements, whole‐cell voltage‐clamp recordings from AII amacrine cells were performed with an EPC10‐triple amplifier (HEKA Elektronik). For experiments with simultaneous dual recording from pairs of synaptically coupled cells, whole‐cell voltage‐clamp recordings from AII amacrine and OFF‐cone bipolar cells were performed with an EPC9‐dual amplifier (HEKA Elektronik). After establishing GΩ‐seals, currents caused by the recording electrode capacitance (*C*_fast_) were automatically measured and neutralized by the amplifier. In the experiments with capacitance recordings, the *C*_fast_ was 4.45 ± 0.44 (SD) pF (range 3.53–5.54 pF) and the average *C*_fast_ time constant was 692 ± 199 (SD) ns (range 309–1297 ns; *n* = 18 cells). After breaking into the cell, currents caused by the cell membrane capacitance (*C*_slow_) were partially neutralized by the amplifier and when we sampled current responses for measuring depolarization‐evoked exocytosis, the *C*_slow_ capacitance neutralization circuitry was always enabled. When we sampled current responses for measuring absolute capacitance values and current responses evoked by ZAP functions (see below), the *C*_slow_ capacitance neutralization circuitry was transiently disabled. Between periods with capacitance measurements, cells were voltage‐clamped at *V*_hold_ = −60 mV. Signals were low‐pass filtered with a corner frequency (−3 dB) at 1/5 of the inverse of the sampling interval (typically 50 *µ*sec). For experiments where we measured depolarization‐evoked exocytosis, linear leak and capacitive currents were subtracted by a P/N protocol, that is, the average leak response (*N* = 5 repetitions) was multiplied by 5 before it was subtracted from the original response.

When we investigated the frequency dependence of activation of voltage‐gated currents in AII amacrine cells, we used a time‐varying voltage stimulus described by a ZAP function (impedance (*Z*) amplitude profile (AP)):

(1) $$V\left(t\right)=a\times \sin\left(bt^{c}+d\right)+V_{\text{hold}}$$

where *a* is the peak amplitude, and *b*, *c*, and *d* are empirically assigned constants (Puil et al., 1986). The values of these constants were chosen to obtain waveforms with peak amplitudes ±15, ±20, ±30 or ±50 mV (relative to *V*_hold_) and frequency ranging from ~5 Hz to ~2.5 kHz. Each waveform had a duration of 1 sec, with constant voltage segments (200 msec duration) added at the beginning and end. Before using a waveform as a stimulus, it was temporally reversed, such that the highest frequencies preceded the lowest frequencies. To subtract linear leak and capacitive currents, we generated leak subtraction stimuli by multiplying the amplitude of each original stimulus waveform by 1/4. The average leak response (*N* = 10 repetitions) was multiplied by 4 and subtracted from the response evoked by the original stimulus.

### Capacitance measurements in physiological recordings

Capacitance measurements were obtained with the “Sine + DC” lock‐in technique (Lindau and Neher 1988; Gillis 1995) as implemented in Patchmaster software in combination with the EPC10 amplifier. For experiments where we measured the increase of capacitance evoked by exocytosis, we used a fixed sine wave frequency of 2 kHz and peak amplitude of ±20 mV relative to *V*_hold_ (Bala‐krishnan et al. 2015). For experiments focused on measuring the absolute capacitance, we applied sine wave voltage stimuli with different frequencies (*f*_sine_) between 100 Hz and 10 kHz and peak amplitude of ±15 mV (relative to *V*_hold_). For a sine wave stimulus with frequency *f*_sine_, the current signal was low‐pass filtered (analog 3‐ and 4‐pole Bessel filters in series) with a cut‐off frequency of 2 × *f*_sine_ and sampled at a frequency of 10 × *f*_sine_. After sampling, the current signal was processed by the lock‐in extension of Patchmaster to obtain estimates of total membrane capacitance (*C*_m_), total membrane conductance (*G*_m_; inverse of total membrane resistance (*R*_m_)) and series conductance (*G*_s_; inverse of series resistance (*R*_s_)) with a temporal resolution of one data point per sine wave cycle. In some graphs we have plotted *G*_m_ instead of *R*_m_, and to be internally consistent we then also plotted *G*_s_ instead of *R*_s_. The reversal potential (*E*_rev_) of the direct current (DC; steady‐state) (*I*_DC_) was by default set to −15 mV.

For measurements of depolarization‐evoked exocytosis, the phase shift and attenuation of the measuring system was calibrated with the “calculated” method. For experiments where the focus was on measuring the capacitance as such and the influence of different sine wave frequencies (see below), we manually calibrated phase shift and attenuation values for each sine wave frequency used in the experiments. This was done by using the 6 pF capacitor of the MC‐10 model cell circuit (HEKA Elektronik). First, the phase shift introduced by the instrumentation was measured (corrected for the 90° phase shift introduced by the model cell capacitor). Next, the capacitance was compensated by the *C*_fast_ capacitance neutralization circuitry of the EPC10‐Patchmaster instrumentation and we verified that the apparent capacitance subsequently measured with the “Sine + DC” lock‐in technique was ~0. The measured capacitance value was then decompensated by 2 pF and the capacitance measurement was repeated. Finally, the attenuation factor was adjusted such that the measured capacitance was equal to the magnitude of the decompensation.

Stimulus‐evoked changes in *C*_m_ (*ΔC*_m_), *R*_m_ (*ΔR*_m_; or the inverse, *ΔG*_m_), and *R*_s_ (*ΔR*_s_; or the inverse, *ΔG*_s_) were calculated as the difference between the average of each parameter during a 1600‐msec period before the stimulus and the average value during a 400‐msec period after the stimulus (*f*_sine_ = 2 kHz). The standard depolarizing stimulus applied to evoke exocytosis from an AII amacrine cell was a pulse from *V*_hold_ = −90 mV to −20 mV. Depolarizing stimuli were applied at intervals of ~60 sec, sufficient to recover from paired‐pulse depression and facilitation of release (Veruki et al. 2006; Balakrishnan et al. 2015). Before and after the depolarizing voltage pulse, the membrane potential was held constant (i.e., without sine wave modulation) for 20 and 100 msec, respectively. In experiments where we only obtained baseline estimates of *C*_m_, *G*_m_, and *G*_s_ at a series of sine wave stimulus frequencies (100 Hz–10 kHz), each data point was calculated as the average of the results from 10 sine wave cycles and before analysis, the waveform was low‐pass filtered at 20 Hz.

### General analysis and data presentation

Data were analyzed with Fitmaster (HEKA Elektronik) and IGOR Pro (WaveMetrics, Lake Oswego, OR). Experimental data are presented as means ± SD (*n* = number of cells or repetitions as stated). The number of individual traces included in the averaged current traces in the figures is stated for each case.

### Wide‐field fluorescence microscopy

In the physiological experiments with capacitance recordings, wide‐field fluorescence microscopy was used to acquire image stacks of AII amacrine cells filled with fluorescent dye, using a TILLvisION system (TILL Photonics, Munich, Germany). An image stack was acquired as a series of optical sections collected at temporal intervals of ~500 msec and focal plane intervals of 0.5 *µ*m, using a cooled, interline transfer CCD camera (Imago QE). The excitation light source (Polychrome V) was coupled to the epifluorescence port of the microscope with a custom‐made condensor via a quartz fiber‐optic light guide. The wavelength of the excitation light was 570 nm and the exposure time was 50–100 msec. At the total magnification used, the pixel size was 106–108 nm in the X‐/Y‐direction. The fluorescence mirror unit (U‐MF2; Olympus) consisted of a dichroic mirror (T585LP), an excitation filter (ET560/40x), and an emission filter (ET630/75m). All filters were purchased from Chroma (Bellows Falls, VT). After acquisition, Huygens Essential (Scientific Volume Imaging, Hilversum, the Netherlands) was used to remove noise and reassign out‐of‐focus light by deconvolution with a theoretical point‐spread function (CMLE method). Maximum intensity projections were generated with Huygens Essential. Final adjustments of contrast, brightness, levels, and gamma were applied homogeneously across the entire image.

### Multiphoton excitation (MPE) fluorescence microscopy and Ca^2+^ imaging

Red (from Alexa 594) and green (from OGB‐1) fluorescence for structural and functional imaging, respectively, were imaged with a ×20 water immersion objective (0.95 NA; Olympus) using a custom‐modified Movable Objective Microscope (Sutter Instrument) equipped with a computer‐controlled, mode‐locked, ultrafast‐pulsed Ti:sapphire laser (Mai Tai DeepSee; SpectraPhysics, Irvine, CA) tuned to 810 nm (for details, see Castilho et al. 2015). For cellular morphology, image stacks were acquired as a series of optical slices (1024 × 1024 pixels; 2 frames/slice) at focal plane intervals of 0.4 *µ*m. MPE microscopy and image acquisition was controlled by ScanImage software (version 3.8.1; Pologruto et al. 2003). For imaging intracellular Ca^2+^ dynamics in processes of AII amacrine cells, we sampled fluorescence (OGB‐1) from constant focal planes in frame‐scan mode (32 × 32 pixels; temporal resolution ~15 Hz).

Ca^2+^ imaging started 10–15 min after establishing the whole‐cell configuration to allow for maximal indicator loading at AII amacrine cell processes. Frame‐scan imaging data were analyzed by custom routines developed under IGOR Pro. Background fluorescence (*F*_b_) was measured as the average signal from a rectangular area close to the region of interest and baseline fluorescence (*F*_0_) was measured by averaging the signal during a ~550 to ~760 msec long interval before stimulus onset. For a given signal (*F*), the relative change in fluorescence related to a change in Ca^2+^ was calculated as (Yasuda et al. 2004):

(2) $$\frac{\Delta F}{F_{0}}=\frac{F-F_{0}}{F_{0}-F_{b}}$$

and for simplicity referred to as *ΔF*/*F*. After Ca^2+^ imaging, a *Z* stack that sampled the complete morphology of the cell was acquired, using the red fluorescence from Alexa 594. Deconvolution of Z stacks for morphology and generation of maximum intensity projections were performed as described above for wide‐field fluorescence imaging.

### Computer simulations

Computer simulations were performed with NEURON (version 7.4) running under Mac OS X (10.9.5) (Carnevale and Hines 2006). Simulations of single neurons were run with a variable time step and the absolute tolerance set to 0.0001. For control, some simulations were repeated after reducing the absolute tolerance to 0.00001, but the results did not change appreciably. For simulations of networks of neurons electrically coupled by gap junctions, we used a fixed time step of 5 *µ*sec. For analysis, data were imported to IGOR Pro and processed with custom routines. In the simulations, an idealized single‐electrode voltage clamp (SEClamp; taken from the standard repertoire of NEURON point processes) was connected to a specific AII amacrine cell compartment, corresponding to the soma or a lobular appendage. For the simulations, we used three representative AII amacrine cells, all belonging to a larger population of cells for which we have previously developed compartmental models (Zandt et al. 2018). These models were developed from correlated morphological reconstructions and physiological measurements, with the best‐fitting passive membrane properties (cytoplasmic resistivity; *R*_i_, specific membrane capacitance; *C*_m_, and specific membrane resistance; *R*_m_) determined for each cell. The best‐fit passive membrane parameters of the three cells are displayed in Table 1. In the simulations, we varied the theoretical value of *R*_s_ for the SEClamp (*R*_s(theory)_) to examine potential effects of uncompensated *R*_s_ on the capacitance measurements. *E*_rev_ of the leak current (e_pas) was set to −60 mV. Spatial discretization (compartmentalization) was implemented by applying the *d_lambda* rule (Carnevale and Hines 2006). The alternating current (AC) length constant at 100 Hz (*λ*_100_) was calculated for each section and the number of segments (*nseg*) in each section was adjusted such that the length of each segment was smaller than a fraction *d_lambda* (set to 0.01 or 0.1) of *λ*_100_. For control, a few simulations were repeated after reducing *d_lambda* to 0.001 and calculating the AC length constant at 1000 Hz (*λ*_100_), but the results did not change appreciably. Before each simulation run, the model was initialized to steady‐state (Carnevale and Hines 2006).

**Table 1:** Best‐fit parameters of AII amacrine cells used for compartmental modeling.

Cell #	*C*_m_ (*µ*F/cm^2^)	*R*_m_ (kΩ cm^2^)	*R*_i_ (Ω cm)
2	0.86	43	223
12	0.87	25	223
13	0.90	36	224

Passive membrane parameters (*C*_m_, *R*_m_, *R*_i_) for the three AII amacrine cells and the corresponding compartmental models used for computational modeling in this study. The numerical identities for the cells correspond to those in the original study by Zandt et al. (2018). The best‐fit parameters were obtained using NEURON's multiple run fitter to directly fit the response of each cell's morphological model to the physiological data obtained for the same cell. The physiological measurements were obtained with whole‐cell voltage‐clamp recording after complete block of gap junction‐mediated electrical coupling with MFA (for details, see Zandt et al. 2018).

To simulate an increase in capacitance, we increased the diameter of a specific section of an AII amacrine cell. Depending on the morphology of the section, the diameter was increased either for all points along the length or only for a limited range, with the goal of restricting the size increase to the structure that appeared as a lobular appendage. For analysis of the capacitance of the computer model, we applied a sine wave voltage stimulus using an SEClamp point process. For single‐cell simulations, the temporal resolution of the sine wave stimulus was 1 *µ*sec. For network simulations, the temporal resolution of the sine wave stimulus was set to 100 points per sine wave cycle. The peak amplitude was ±15 mV (relative to *V*_hold_) and *f*_sine_ ranged from 100 Hz–10 kHz (100, 200, 400, 1000, 2000, 4000, 5000, and 10,000 Hz; unless otherwise stated).

For the analysis, we first determined the phase of the sine wave stimulus by fitting it with the function,

(3) $$V\left(t\right)=A\times \sin\left(2\pi f_{\text{sine}}t+\alpha \right)+V_{\text{hold}}$$

where *A* is the amplitude, *α* is the phase (in radians), and 2*πf*_sine_ is equivalent to the angular frequency (*ω*). Second, we determined the real and imaginary components of the current response (in phase and 90° out of phase with the voltage stimulus, respectively) by fitting it with the function,

(4) $$I\left(t\right)=A_{1}\times \sin\left(2\pi f_{\text{sine}}t+\alpha \right)+A_{2}\times \cos\left(2\pi f_{\text{sine}}t+\alpha \right)+I_{\text{DC}}$$

where *A*_1_ is the amplitude of the real component, *A*_2_ is the amplitude of the imaginary component, *α* is the phase (determined by equation (3)), and *I*_DC_ is the steady‐state (holding) current. *C*_m_, *R*_m_, and *R*_s_ were calculated from *A*_1_, *A*_2_ and *I*_DC_, according to equation (28) in Gillis (1995):

$$C_{m}=\frac{1}{\omega B}\frac{{\left(A^{2}+B^{2}-AG_{t}\right)}^{2}}{{\left(A-G_{t}\right)}^{2}+B^{2}}$$

$$R_{m}=\frac{1}{G_{t}}\frac{{\left(A-G_{t}\right)}^{2}+B^{2}}{A^{2}+B^{2}-AG_{t}}$$

(5) $$R_{s}=\frac{A-G_{t}}{A^{2}+B^{2}-AG_{t}}$$

where *A* and *B* are the amplitudes of the real (*A*_1_) and imaginary (*A*_2_) components (obtained from equation (4)) normalized to the amplitude of the voltage sinusoid stimulus and *G*_t_ = *I*_DC_/(*V*_hold_–*E*_rev_) (Gillis 1995). By default, *E*_rev_ for *I*_DC_ was set to −60 mV, corresponding to *E*_rev_ (e_pas) of the leak current in the simulated cell.

### Computer simulations of electrical synapses between compartmental models of AII amacrine cells

To implement electrical coupling in the AII compartmental models, we placed identical copies of the same cell in a hexagonal network and linked each cell to all of its nearest (directly connected) neighbors. Because we did not have reconstructions of cells that were connected to each other in situ, the electrical synapses were implemented between nontouching points on the cells. To determine the locations of the contact points, we first calculated the cell's center of mass as a 3D coordinate (*X*, *Y*, *Z*) that represented all the cell's reconstruction points. The center of mass 3D coordinate was then projected (along the *Y* axis, corresponding to the long axis of the cell) onto the *XZ* plane. The resulting point defined the origin of an angle *θ* (set to 120°) that again defined a specific sector within which electrical synapses between the cell and one of its neighbors were implemented. The sector was extruded through the image stack along the *Y* axis (as a “cookie cutter”), effectively generating the volume of a pie slice. For both cells of a given pair, *N* reconstruction points were randomly selected within the specified sector volume. To obtain identical networks for different simulations, the same seed of the random number generator was used to instantiate the network for each simulation. After selecting *N* points for the two cells of a given pair, the points were sorted by height (position along the *Y* axis) such that the contact points corresponding to electrical synapses connected processes at comparable heights. We also restricted the height (range along the *Y* axis) where contacts could be made, corresponding overall to homologous gap junctions between AII amacrine cells and heterologous gap junctions between AII amacrine cells and ON‐cone bipolar cells in strata S3 (the most proximal part), S4 and S5 of the inner plexiform layer (Strettoi et al. 1992; Chun et al. 1993).

For the simulations reported here, *N* was set to 20 for each cell pair. This number was obtained from manual counting of visually identified contact points (potentially corresponding to gap junction contacts) between pairs of electrically coupled AII amacrine cells, verified by dual, simultaneous whole‐cell recording where we measured the junctional conductance betwen the two cells (Veruki and Hartveit, 2002a). The cells of each pair were filled with fluorescent dyes during whole‐cell recording (Alexa 488 in one cell and Alexa 594 in the other cell) and imaged with MPE microscopy (Zandt et al. 2017). For three cell pairs, the number of contact points was 20, 22 and 25, respectively. The junctional conductance of an individual contact point was set to the total pairwise junctional conductance divided by the number of contacts between two neighboring cells. For example, if the pairwise junctional conductance was set to 700 pS (Veruki and Hartveit 2002a), the conductance of each individual contact was set to 35 pS (700 pS/20 contacts). All simulations of gap junction‐coupled networks were run with a network of 25 cells, arranged in a 5 × 5 trapezoidal grid (see Results). With this arrangement, a single cell made a total of 120 gap junction contacts with its six nearest neighbors. The stimuli were applied to the cell in the middle of the network and the simulations were run with “open boundary” conditions, that is, with the cells at the edges of the network connected with electrical synapses to resting membrane potential.

## Results

### Depolarization‐evoked exocytosis from AII amacrine cells in adult rat retina

Exocytosis of synaptic vesicles from the lobular appendages of AII amacrines is expected to increase the membrane surface area, and consequently the membrane capacitance, when the vesicles fuse with the cell membrane (Balakrishnan et al. 2015). However, because an AII amacrine has an elaborate dendritic tree that cannot be represented by an electrically equivalent simple RC‐circuit, it is unclear to which extent exocytosis occurring at different locations in the cell can be detected and how this is reflected in changes of the apparent capacitance measured with a lock‐in amplifier. To investigate this, we first performed physiological experiments with AII amacrines in rat retinal slices, including capacitance measurements similar to those performed by Balakrishnan et al. (2015) for AII amacrines in mouse retinal slices.

**Figure 1:**
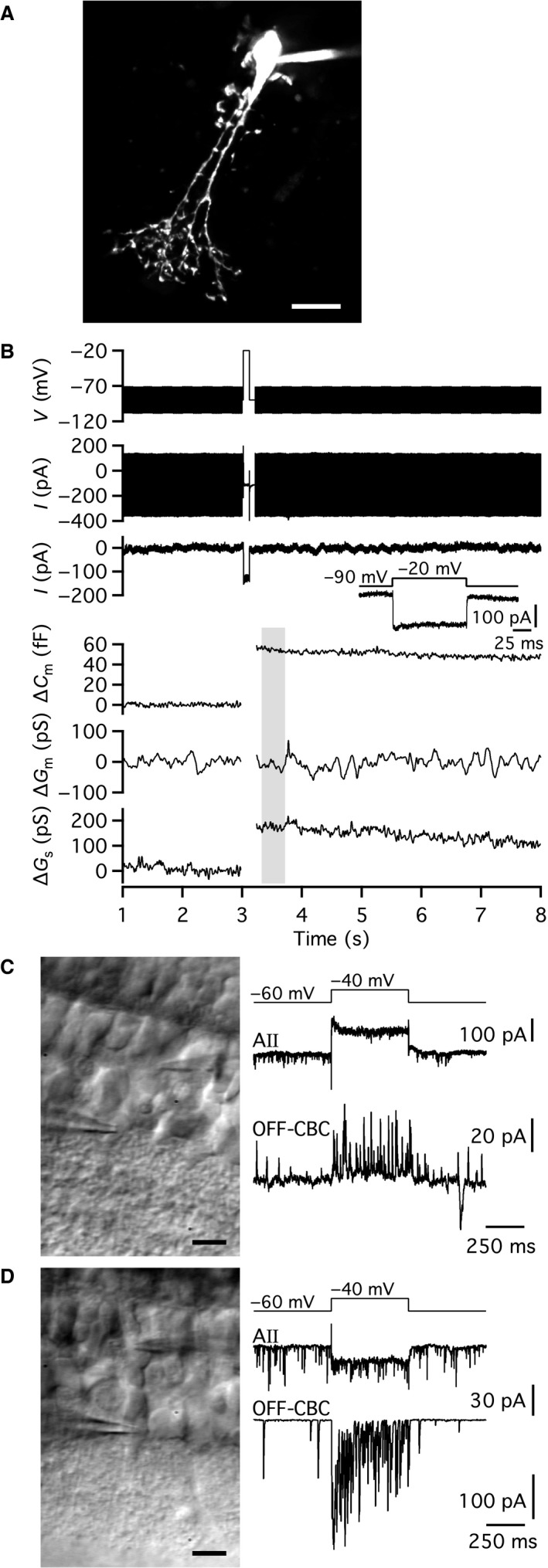
Physiological evidence for depolarization‐evoked exocytosis of neurotransmitter from AII amacrine cells in rat retinal slices. (A) AII amacrine cell filled with Alexa 594 via patch pipette during whole‐cell recording. Maximum intensity projection (MIP; along *Z* axis) generated from wide‐field fluorescence image stack after deconvolution. (B) Using the “Sine + DC” technique to measure exocytosis‐evoked capacitance increase in whole‐cell soma recording of AII amacrine cell in retinal slice (same cell as in A). Sine wave stimulation (2 kHz; ±15 mV from *V*_hold_ = −90 mV; top) before and after a 100 msec depolarization to −20 mV to activate voltage‐gated Ca^2+^ current and Ca^2+^‐dependent exocytosis. Top and bottom current traces show currents evoked by the sine wave stimulus and depolarizing pulse without and with leak subtraction, respectively. Inset shows inward Ca^2+^ current at higher time resolution. For each sine wave cycle, one data point was obtained for cell capacitance (*C*_m_), cell membrane conductance (*G*_m_), and series conductance (*G*_s_) and the data are displayed after baseline subtraction (*ΔC*_m_, *ΔG*_m_, and *ΔG*_s_). Note that the depolarization‐evoked increase of *ΔC*_m_ (~60 fF) is accompanied by an increase of *ΔG*_s_, but not by a change in *ΔG*_m_. (C) Simultaneous, dual recording of an AII amacrine cell and an OFF‐cone bipolar cell synaptically connected to each other. Infrared differential interference contrast (IR‐DIC) videomicrograph (left) shows recording pipettes and retinal slice during whole‐cell recording. Traces (right) illustrate depolarizing voltage‐clamp stimulus waveform applied to AII amacrine cell (top) and corresponding presynaptic response in AII amacrine cell (middle; AII) and postsynaptic response with outward currents in OFF‐cone bipolar cell (bottom; OFF‐CBC). Because the OFF‐cone bipolar cell was voltage‐clamped at *V*_hold_ = −60 mV and the chloride equilibrium potential (*E*_Cl_) was ~−90 mV, a chloride current will appear as an outward current. (D) Simultaneous, dual recording of an AII amacrine cell and an OFF‐cone bipolar cell synaptically connected to each other. IR‐DIC videomicrograph (left) shows recording pipettes and retinal slice during whole‐cell recording. Traces (right) illustrate depolarizing voltage‐clamp stimulus waveform applied to AII amacrine cell (top) and corresponding presynaptic response in AII amacrine cell (middle; AII) and postsynaptic response with inward currents in OFF‐cone bipolar cell (bottom; OFF‐CBC). Because the OFF‐cone bipolar cell was voltage‐clamped at *V*_hold_ = −60 mV and the chloride equilibrium potential (*E*_Cl_) was ~0 mV, a chloride current will appear as an inward current. Scale bars: 10 *µ*m (A, C, D).

To measure the putative depolarization‐evoked increase of capacitance caused by exocytosis at lobular appendages of AII amacrine cells, we applied 100 msec voltage pulses from −90 mV (*V*_hold_) to −20 mV (*V*_com_). The capacitance was measured using a 2 kHz sine wave (±20 mV relative to *V*_hold_), applied before and after the voltage pulse (cf. Balakrishnan et al. 2015). For the cell illustrated in Figure 1A, the voltage pulse evoked an inward current (apparent after subtracting the linear leak and uncompensated capacitive currents) and an associated increase of capacitance (Fig. 1B). The depolarization‐evoked inward current is illustrated at higher time resolution in the inset of Figure 1B. With a sine wave frequency of 2 kHz, the average baseline capacitance (*C*_m_) was 7.58 pF (averaged over 1600 msec) and the average *C*_m_ after the depolarization was 7.63 pF (averaged over 400 msec), corresponding to a *ΔC*_m_ of ~55 fF (*n* = 3 repetitions). For the same cell, there was little change of *G*_m_ (*ΔG*_m _~0; Fig. 1B). The baseline value of *G*_s_ was ~63.2 nS (*R*_s_ = 15.823 M*Ω*) and after the depolarization the value of *G*_s_ was transiently elevated (Fig. 1B), with a maximum value of ~63.4 nS (*R*_s_ = 15.773 M*Ω*), corresponding to *ΔG*_s_ = 173 pS (*ΔR*_s_ ~0.05 M*Ω*). When depolarizing stimuli were repeated every 60 sec, the evoked increase of capacitance was initially robust, but started to run down shortly after the whole‐cell recording configuration had been established. For the cell illustrated in Figure 1, the depolarization‐evoked *ΔC*_m_ was reduced to ~20 fF after ~15 min recording time. Importantly, the magnitude of *ΔG*_s_ also ran down, in parallel with the time‐dependent reduction of *ΔC*_m_, suggesting that it resulted from exocytosis and cross‐talk between *ΔG*_s_ and *ΔC*_m_ (Gillis, 1995; Hallermann et al. 2003; Oltedal and Hartveit, 2010). For five AII amacrine cells tested in the same way, the average *ΔC*_m_ was 49.4 ± 3.2 fF (range 47.3–54.7 fF; *n* = 3–5 repetitions for each cell). In addition to AII amacrine cells, we also recorded from two wide‐field amacrine cells with morphology similar to those reported in Veruki et al. (2007). In neither of the wide‐field amacrine cells did we observe a depolarization‐evoked change in capacitance (data not shown).

If the depolarization‐evoked increase of capacitance of AII amacrine cells reflects exocytosis of glycine‐containing synaptic vesicles at the lobular dendrites of these cells, it should be possible to observe corresponding postsynaptic responses mediated by chloride currents in OFF‐cone bipolar cells. To investigate this, we performed simultaneous paired recordings between AII amacrine cells and OFF‐cone bipolar cells in retinal slices. In the paired recording illustrated in Figure 1C, the AII amacrine cell was depolarized from −60 mV (*V*_hold_) to −40 mV (*V*_com_) for 500 msec. The OFF‐cone bipolar cell was voltage‐clamped at −60 mV. The depolarization of the AII amacrine evoked a distinct postsynaptic response in the OFF‐cone bipolar cell, with increased frequency of partially overlapping outward postsynaptic currents (PSCs). With *E*_Cl_ ~−90 mV, a chloride current (through glycine receptor channels) will appear as an outward current at *V*_hold_ = −60 mV, which is consistent with the experimental observations. Similar results were seen for seven other cell pairs. Because the synaptic response ran down relatively quickly, most likely caused by rundown of exocytosis in the AII amacrine, we did not attempt to examine the PSC pharmacologically to verify that it was mediated by activation of glycine receptors. Instead, we repeated dual‐recording experiments with a higher chloride concentration in the pipette solution of the OFF‐cone bipolar cell, corresponding to *E*_Cl _~0 mV. For this condition, depolarization of the AII amacrine from −60 mV (*V*_hold_) to −40 mV (*V*_com_) for 500 msec evoked an inward PSC in the OFF‐cone bipolar cell (*V*_hold_ = −60 mV; Fig. 1D), consistent with a chloride current. Similar responses were seen for three other cell pairs. These results strongly suggested that depolarizing voltage steps that activate a voltage‐gated Ca^2+^ current in AII amacrine cells (cf. Habermann et al. 2003) also evoke exocytosis of synaptic vesicles (at the lobular dendrites).

### Measuring the capacitance of AII amacrine cells with the “Sine + DC” technique: simulations with compartmental models and physiological recordings from cells in retinal slices

Physiological measurements of depolarization‐evoked exocytosis as an increase of capacitance using the “Sine + DC” technique requires the selection of a specific sine wave frequency. As a first step in selecting an optimal frequency, we investigated how the magnitude of the apparent *C*_m_ (relative to the true *C*_m_) depends on the sine wave frequency, using computer simulations with compartmental models of AII amacrine cells (Zandt et al. 2018). We started by asking how the frequency of the sine wave stimulus influences the measurement of *C*_m_, *R*_m_ and *R*_s_ and how *R*_s_ of the voltage clamp influences the measurements of *C*_m_, *R*_m_ and *R*_s_ for a given sine wave frequency.

**Figure 2:**
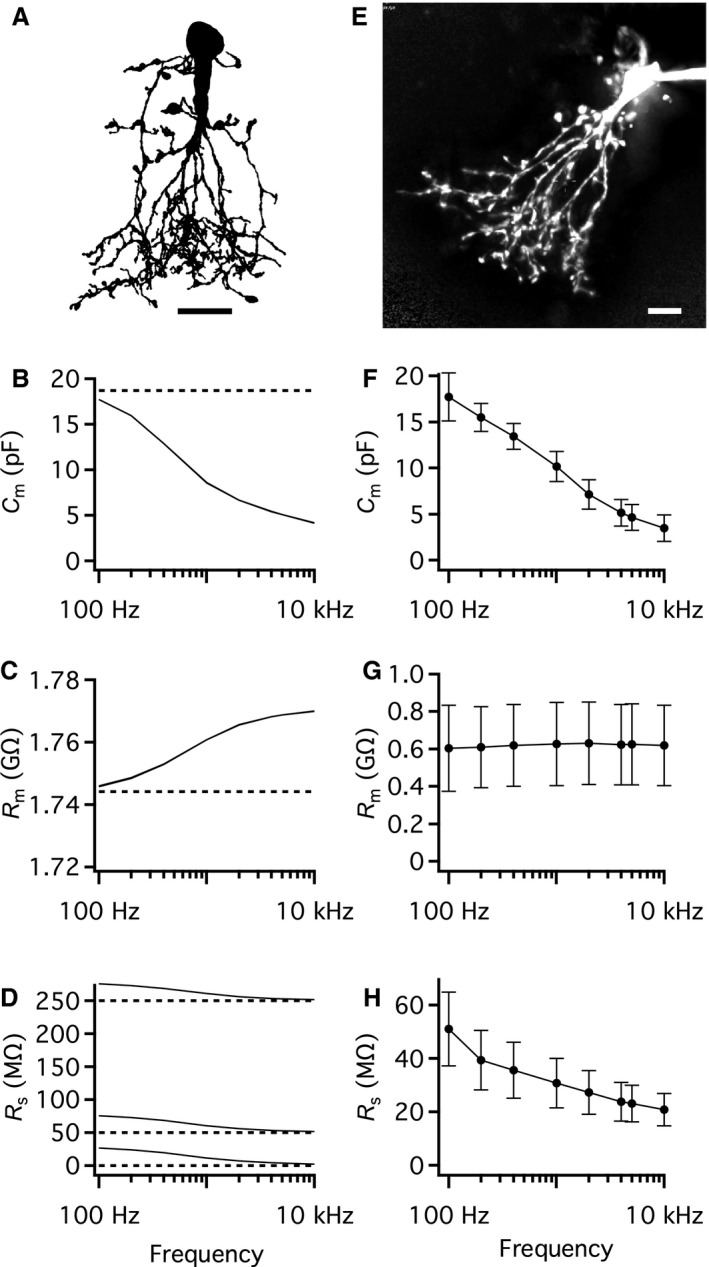
Performance of the “Sine + DC” technique for measuring capacitance of AII amacrine cells with computer simulations and physiological whole‐cell recording. (A) Shape plot of morphologically reconstructed AII amacrine cell (for details, see Zandt et al. 2017) used for computer simulations. (B–D) Estimates of total capacitance (*C*_m_; B), total membrane resistance (*R*_m_; C), and series resistance (*R*_s_; D) as a function of sine wave stimulation frequency (100 Hz–10 kHz) for three different values of *R*_s_ (*R*_s(theory)_; 1, 50, and 250 M*Ω*). Here and later, theoretical parameter values indicated by broken horizontal lines (*C*_m_ = 18.69 pF, *R*_m_ = 1.744 G*Ω*). Simulations performed in *NEURON* software with idealized single‐electrode voltage clamp (SEClamp) connected to the soma (B–D). In all simulations, the sine wave voltage stimulus amplitude was ±15 mV (from *V*_hold_ = −80 mV). (E) AII amacrine cell filled with Alexa 594 via patch pipette during whole‐cell recording. MIP (along *Z* axis) generated from wide‐field fluorescence image stack after deconvolution. (F–H) Estimates of total capacitance (*C*_m_; F), total membrane resistance (*R*_m_; G), and series resistance (*R*_s_; H) as a function of sine wave stimulation frequency (100 Hz–10 kHz) during whole‐cell recording of AII amacrine cells (*n* = 9 cells, including cell in E). Data points represent mean ± SD. Sine wave amplitude ± 15 mV from *V*_hold_ = −80 mV. Here and later, the estimates obtained with physiological recordings set the *E*_rev_ of *I*_DC_ to −15 mV. For each sine wave stimulus frequency used, manual calibration of the patch‐clamp amplifier was performed with an external capacitor (see Methods). Scale bars: 10 *µ*m (A and E).

For a round cell that can be modeled as a simple RC‐circuit, an optimal range of sine wave stimulus frequencies can be directly calculated (equation (52) in Gillis (1995)). This approach cannot be used, however, for a neuron with a branching dendritic tree that cannot be adequately described by a single‐compartment equivalent electrical circuit. Instead, we first used computer simulations to systematically vary the frequency (from 100 Hz–10 kHz) of the sine wave voltage‐clamp stimulus and calculated the apparent capacitance from the voltage stimulus and the evoked current. The AII amacrine compartmental model used for the simulations (Fig. 2A) was developed after blocking gap junction coupling pharmacologically (Zandt et al. 2018). It had a specific membrane capacitance of 0.90 *µ*F/cm^2^, a specific membrane resistance of 36 k*Ω* cm^2^ and a cytoplasmic resistivity of 224 *Ω* cm (Table 1; Zandt et al. 2018). For each sine wave frequency, we obtained estimates of *C*_m_, *R*_m_ and *R*_s_ (Fig. 2B–D). We also varied the theoretical value of the *R*_s_ of the voltage clamp (*R*_s(theory)_; 1, 50, and 250 M*Ω*) for each frequency. From the morphological reconstruction, the total membrane surface area was estimated as 2073 *µ*m^2^. These values corresponded to a total membrane capacitance (*C*_m(theory)_) of ~18.7 pF and a membrane resistance (*R*_m(theory)_) of ~1.744 GΩ. As illustrated in Figure 2B, the estimate of *C*_m_ depended strongly on sine wave frequency, with increasing frequency causing progressive and pronounced underestimation of *C*_m_. Under these ideal conditions, with no noise, the different values of *R*_s(theory)_ had no effect (the three curves completely overlap; Fig. 2B). The estimate of *R*_m_ was also frequency‐dependent, with increasing overestimation for increasing sine wave frequencies, but the accuracy (defined as *R*_m_/*R*_m(theory)_) was relatively high throughout the range of frequencies (Fig. 2C). The different values of *R*_s(theory)_ had no effect on the estimates of *R*_m_ (Fig. 2C). *R*_s_ was estimated with high accuracy at high sine wave frequencies, but at lower sine wave frequencies the accuracy markedly decreased, with increasing overestimation for decreasing frequency values (Fig. 2D). This pattern was the same for all three values of *R*_s(theory)_.

We next compared the capacitance measurements based on computer simulations (Fig. 2B–D) to the capacitance measurements based on physiological recordings of AII amacrine cells in slices (*n* = 9 cells; *V*_hold_ = −80 mV; peak amplitude ±15 mV; Fig. 2E–H). As we observed for computer simulations, the estimates for *C*_m_ (Fig. 2F) and *R*_s_ (Fig. 2H) were reduced when we increased the sine wave frequency from 100 Hz–10 kHz. In contrast, the estimates for *R*_m_ were essentially independent of frequency (Fig. 2G). These estimates were based on the default value of *E*_rev_ for *I*_DC_ (−15 mV) when recording with a Cs^+^‐based intracellular solution. The values for *R*_m_ in our physiological recordings were much lower than the values for *R*_m_ in the compartmental modeling, reflecting the fact that the models were developed from physiological measurements performed after blocking gap junction coupling pharmacologically (Zandt et al. 2018). The influence of gap junction coupling is examined in more detail below.

Taken together, these results strongly suggested that the influence of sine wave frequency on the estimates for *C*_m_ and *R*_s_ in physiological recordings (Fig. 2F and H) did not result from properties of the instrumentation. First, the frequency dependence was very similar to that observed for computer simulations using compartmental models based on morphologically reconstructed AII amacrine cells (Fig. 2B and D). Second, we manually calibrated the phase shift and attenuation values for all sine wave frequencies employed for physiological measurements. When we used the same calibrations to estimate the capacitance of an electronic model cell (simple RC‐circuit), corresponding to an unbranched round cell, there was no evidence for any frequency dependence. The results indicate that when the experimental goal is to measure the absolute value of the membrane capacitance of a complex and branching neuron, like the AII amacrine cell, only a low sine wave frequency (e.g. ~100 Hz) will provide an estimate with high accuracy. The accuracy drops with increasing frequency and is only ~0.5 at ~1 kHz. Importantly, however, this does not directly predict the accuracy of measurements of changes in capacitance evoked by exocytosis and the resulting increase of membrane surface area at discrete locations in the dendritic tree of an AII amacrine cell, as explored in detail below.

### Influence of gap junction coupling on capacitance measurements of AII amacrine cells in physiological recordings

**Figure 3:**
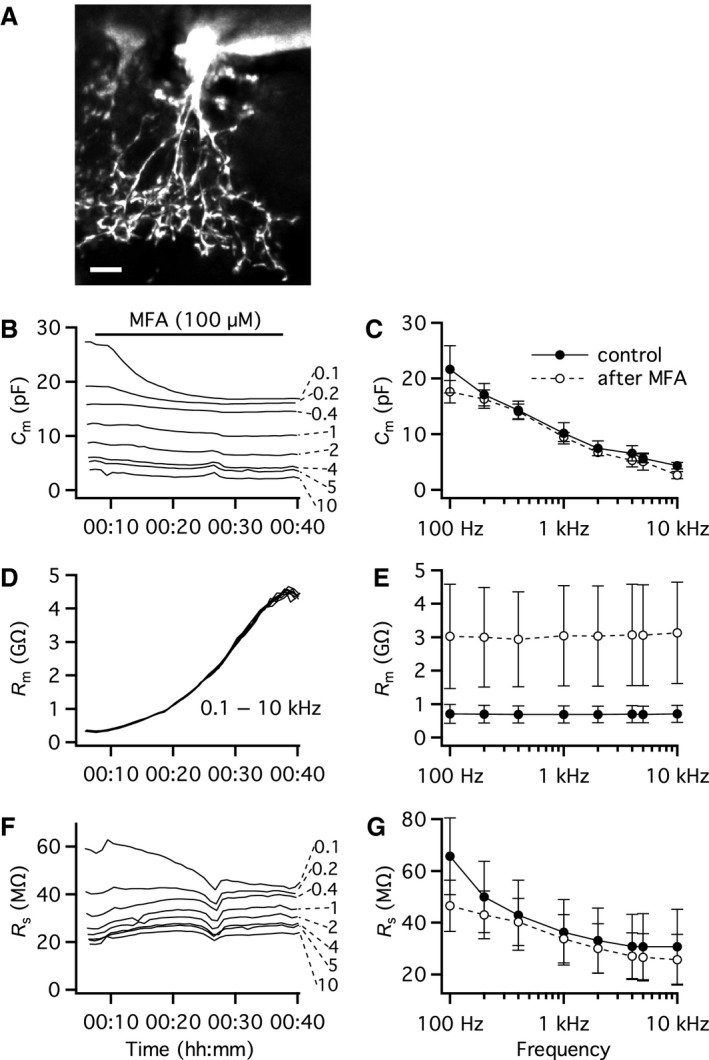
Influence of gap junction coupling on performance of the “Sine + DC” technique for measuring capacitance of AII amacrine cells with whole‐cell recording in physiological experiments. (A) AII amacrine cell filled with Alexa 594 via patch pipette during whole‐cell recording. MIP (along *Z* axis) generated from wide‐field fluorescence image stack after deconvolution. Fluorescent shadow to the left of the cell resulted from a failed attempt at a dual recording. (B) Estimates of total capacitance (*C*_m_) as a function of time for different sine wave stimulation frequencies (0.1–10 kHz; as indicated for each trace) during whole‐cell recording of AII amacrine cell (same cell as in A). Time zero corresponds to breaking into the cell and establishing the whole‐cell recording configuration. To block gap junction coupling, meclofenamic acid (MFA; 100 *µ*mol/L) was applied in the extracellular bath solution, as indicated by the horizontal line. Sine wave amplitude ±15 mV from *V*_hold_ = −80 mV (B–G). (C) Estimates of *C*_m_ as a function of sine wave stimulation frequency (0.1–10 kHz) during whole‐cell recording of AII amacrine cells (*n* = 4 cells; recordings as in B). For each cell, measurements were obtained for the control condition (continuous line; C, E, G), with intact gap junction coupling, and after blocking coupling with MFA (broken line; C, E, G). Data points represent mean ± SD (C, E, G). (D) Estimates of total membrane resistance (*R*_m_) for AII amacrine in A (as in B). (E) Estimates of *R*_m_ as a function of sine wave stimulation frequency (0.1–10 kHz; same AII amacrine cells as in C). (F) Estimates of series resistance (*R*_s_) for AII amacrine in A (as in *B*). (G) Estimates of *R*_s_ as a function of sine wave stimulation frequency (0.1–10 kHz; same AII amacrine cells as in C). Note how the estimates of *C*_m_ (B, C) and *R*_s_ (F, G) are influenced by the sine wave stimulation frequency and how blocking gap junction coupling influences the estimates of *C*_m_ (and to some extent *R*_s_) for lower (100 Hz), but not higher (10 kHz) sine wave stimulation frequencies. The estimates of *R*_m_ are not influenced by the sine wave stimulation frequency (D, E) but gradually increase during block of gap junction coupling by MFA (D). Scale bar: 10 *µ*m (A).

In our first physiological recordings with “Sine + DC” measurement of the capacitance of AII amacrine cells, we did not block gap junction coupling between AII amacrine cells and between AII amacrines and ON‐cone bipolar cells. In previous work from our laboratory with dual recording of pairs of electrically coupled AII amacrine and ON‐cone bipolar cells (Veruki and Hartveit 2002a, 2002b), as well as with dual recording of pairs of AII amacrine cells where artificial electrical coupling was introduced by dynamic clamp electrophysiology (Veruki et al. 2008), we found that sine wave frequencies ≤100 Hz can be effectively transferred between directly coupled cells, depending on the magnitude of the junctional conductance. For higher sine wave frequencies, the attenuation within the cell (from the soma to the gap junctions located in the arboreal dendrites) and across the gap junctions will eventually become sufficiently strong that neighboring electrically coupled cells will no longer contribute to the responses evoked by stimulating an individual cell. To explore this with physiological measurements, we performed whole‐cell recordings and used the “Sine + DC” lock‐in technique to measure the apparent values of *C*_m_, *R*_m_, and *R*_s_ with a range of sine wave frequencies (100 Hz–10 kHz), first in the control condition and then during gradual block of electrical coupling after adding MFA (100 *µ*mol/L) to the bath solution. For the AII amacrine cell illustrated in Figure 3A, we obtained such measurements repeatedly over a period of ~40 min. Following addition of MFA to the bath, the total capacitance estimated with a sine wave frequency of 100 Hz was reduced, decreasing gradually from an initial value of ~27 pF to a final value of ~17 pF during a period of 20–30 min (Fig. 3B). This duration is consistent with the time for MFA to completely block the gap junction coupling of AII amacrine cells (Veruki and Hartveit 2009). For a sine wave frequency of 200 Hz, the reduction of *C*_m_ was smaller (from ~19 to ~16 pF) and for higher‐frequency sine wave stimuli no reduction was observed (Fig. 3B). For three additional cells tested in the same way, we also observed a reduction of total capacitance at 100 Hz, but not at higher frequencies. Figure 3C illustrates the apparent values of *C*_m_ (as a function of sine wave frequency) obtained during the control period with presumed intact gap junction coupling (filled circles and continuous line), and after complete block of coupling with MFA (open circles and broken line; *n* = 4 cells).

For the corresponding estimates of *R*_m_, we observed a gradual increase consistent with gradual block of electrical coupling (Veruki and Hartveit 2009), but there was no influence of the sine wave frequency (Fig. 3D and E). For the estimates of *R*_s_, we observed an influence of the sine wave frequency throughout the recording, both during the initial control period and during block of coupling by MFA, with lower values of *R*_s_ obtained for higher sine wave frequencies (Fig. 3F and G). The estimates obtained for the highest sine wave frequencies displayed little change during block of gap junction coupling, but for the sine wave frequency of 100 Hz, a stronger reduction of *R*_s_ could be observed (Fig. 3F and G).

### Measuring capacitance changes with the “Sine + DC” technique following spatially discrete increases of membrane surface area of AII compartmental models

Our recent investigation of the frequency dependence of attenuation from the soma through the dendritic tree (Zandt et al. 2018), suggests that the ability to detect exocytotic capacitance increase (*ΔC*_m_) with high accuracy will vary as a function of the location at which the increase occurs. Furthermore, because different lobular appendages are located at different anatomical and electrotonic distances from the cell body (where the voltage‐clamp electrode is located), it is likely that the optimal sine wave frequency will vary for different lobular appendages.

**Figure 4:**
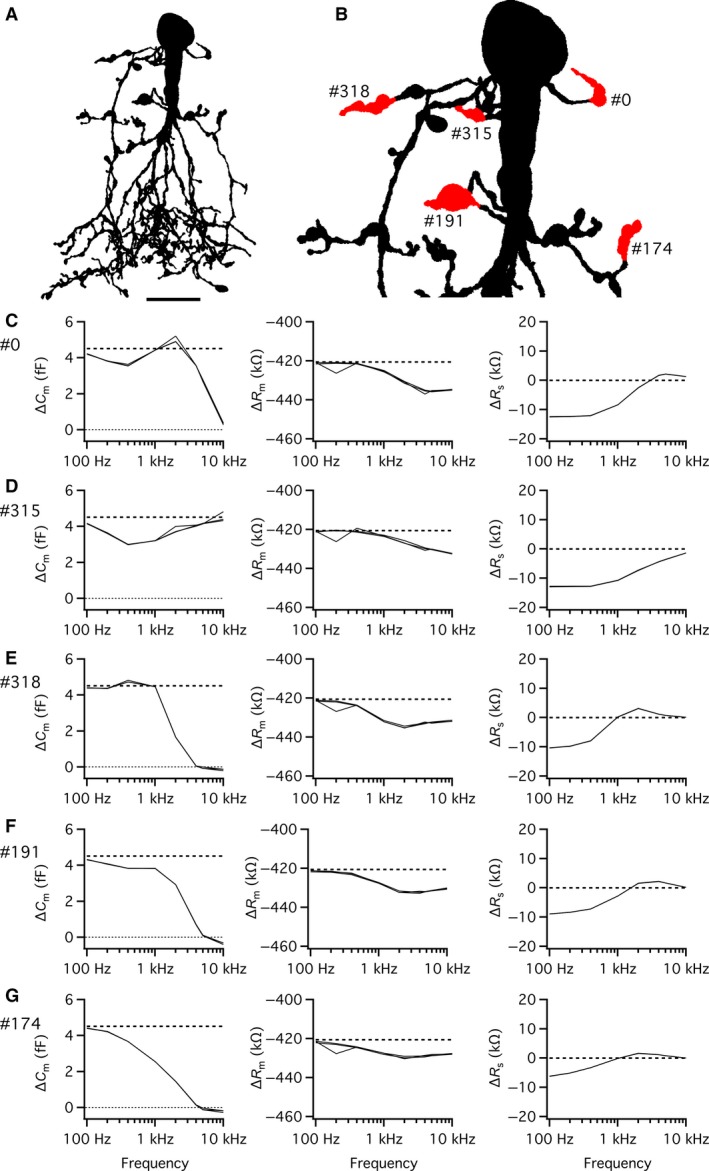
Performance of the “Sine + DC” technique for measuring capacitance increase at different lobular appendages of an AII amacrine cell with computer simulations of soma recording. (A) Shape plot of morphologically reconstructed AII amacrine cell used for computer simulations. (B) Enlarged shape plot of same AII cell restricted to region with soma and lobular dendrites and appendages. Here and later, each lobular dendrite for which an increase of surface area and capacitance was simulated is marked by the section number assigned during *NEURON* simulation and the specific region where the surface was increased is labeled red. (C–G) Estimates of changes in capacitance (*ΔC*_m_; left column), membrane resistance (*ΔR*_m_; middle column), and series resistance (*ΔR*_s_; right column) as a function of sine wave stimulation frequency (100 Hz–10 kHz) after increasing the membrane capacitance of a lobular appendage (indicated by section number to the left) by 4.51 fF (corresponding to an increase of surface area of 0.5 *µ*m^2^ with specific capacitance 0.9016 *µ*F/cm^2^). The rows (corresponding to different lobular appendages) are arranged in order of increasing anatomical and electrotonic distance from soma. Estimates indicated as a function of sine wave stimulation frequency (100 Hz–10 kHz) for three different values of *R*_s_ (*R*_s(theory)_). Simulations performed with SEClamp connected to the soma, in all simulations the sine wave voltage stimulus amplitude was ±15 mV (from *V*_hold_ = −80 mV; C–G). For clarity, the zero line has been indicated by a dotted line in some panels. Note the marked influence of sine wave frequency and location of lobular appendage on accuracy of estimate of *ΔC*_m_ (C–G). Scale bar: 10 *µ*m (A).

Exocytosis at individual lobular appendages was simulated by increasing the capacitance at specific locations in compartmental models of AII amacrines and the capacitance change was estimated with the “Sine + DC” lock‐in technique. For each AII compartmental model examined, an increase in capacitance (*ΔC*_m(theory)_) was simulated as an increase in the membrane surface area of 0.5 *µ*m^2^, corresponding to an increase of approximately 5 fF, depending on the exact value of the specific membrane capacitance for a given cell. With the diameter for glycine‐containing synaptic vesicles used by Balakrishnan et al. (2015) and the specific membrane capacitance values for our compartmental models, this corresponds approximately to 100 vesicles. For the cell illustrated in Figure 4A (with specific membrane capacitance of 0.902 *µ*F/cm^2^), an increase of 0.5 *µ*m^2^ corresponded to *ΔC*_m(theory)_ = 4.51 fF. Each simulation trial corresponded to an increase of capacitance at a single lobular appendage. For the three AII amacrine compartmental models tested, we investigated a total of 6, 12, and 18 different lobular appendages, respectively. For the cell shown in Figure 4A, we have illustrated the results obtained for five different lobular appendages, indicated in the magnified shape plot of Figure 4B. The accuracy, defined as *ΔC*_m_/*ΔC*_m(theory)_, was consistently high for a sine wave of 100 Hz, the lowest frequency tested (Fig. 4C–G; left panels). For higher sine wave frequencies, the results differed, depending on the location of the lobular appendage in the dendritic tree (Fig. 4C–G). For some lobulars, located close to the soma, the accuracy first dropped slightly (for frequencies between 200 and 400 Hz) and then increased again to a local maximum (between 1 and 10 kHz; Fig. 4C and D). For other lobulars, located further from the soma, the accuracy remained relatively high for frequencies between 100 Hz and 1 kHz, followed by a decrease for frequencies ≥1 kHz (Fig. 4E and F). Finally, for lobulars located even further from the soma, the accuracy decreased monotonically from the maximum at 100 Hz and could be ≤50% already at 1 kHz (Fig. 4G). Most likely, the low accuracy at the highest frequencies simply corresponds to the reduction of signal amplitude. The results obtained for the different values of *R*_s(theory)_ were very similar and in several cases overlapped completely (Fig. 4C–G). Qualitatively similar results were obtained when we repeated the simulations with a much larger (and unrealistic) increase of surface area (10 *µ*m^2^; data not shown). These results confirm that the ability to accurately detect exocytotic capacitance increases depends on location in the dendritic tree.

Because the dendritic trees of AII amacrine cells display extensive branching, it is expected that an increase in capacitance may evoke correlated (parallel or antiparallel) changes in the resistive circuit parameters *R*_m_ and/or *R*_s_ when analyzed with the “Sine + DC” technique (Fig. 1B; Gillis 1995; see also Hallermann et al. 2003 and Oltedal and Hartveit 2010). In our simulations, we observed a small decrease of *R*_m_ for all sine wave frequencies (Fig. 4C–G; middle panels). For the lowest frequencies (100–400 Hz), the decrease of *R*_m_ was fully explained by the addition of 0.5 *µ*m^2^ of membrane with specific membrane resistance identical to the rest of the cell membrane. For higher frequencies, the decrease was slightly larger (10–20 k*Ω*; Fig. 4C–G; middle panels). In addition, we consistently observed cross‐talk between *ΔC*_m_ and *ΔR*_s_, with the increase of *C*_m_ accompanied by a small, apparent decrease of *R*_s_ (~10 k*Ω*) for the lower sine frequencies (Fig. 4C–G; right panels). The magnitude of *ΔR*_s_ decreased with increasing sine wave frequency and could reverse to a small increase for a range of higher frequencies (~1–10 kHz; Fig. 4C–G; right panels).

### Relationship between the magnitude of implemented and measured capacitance increase

**Figure 5:**
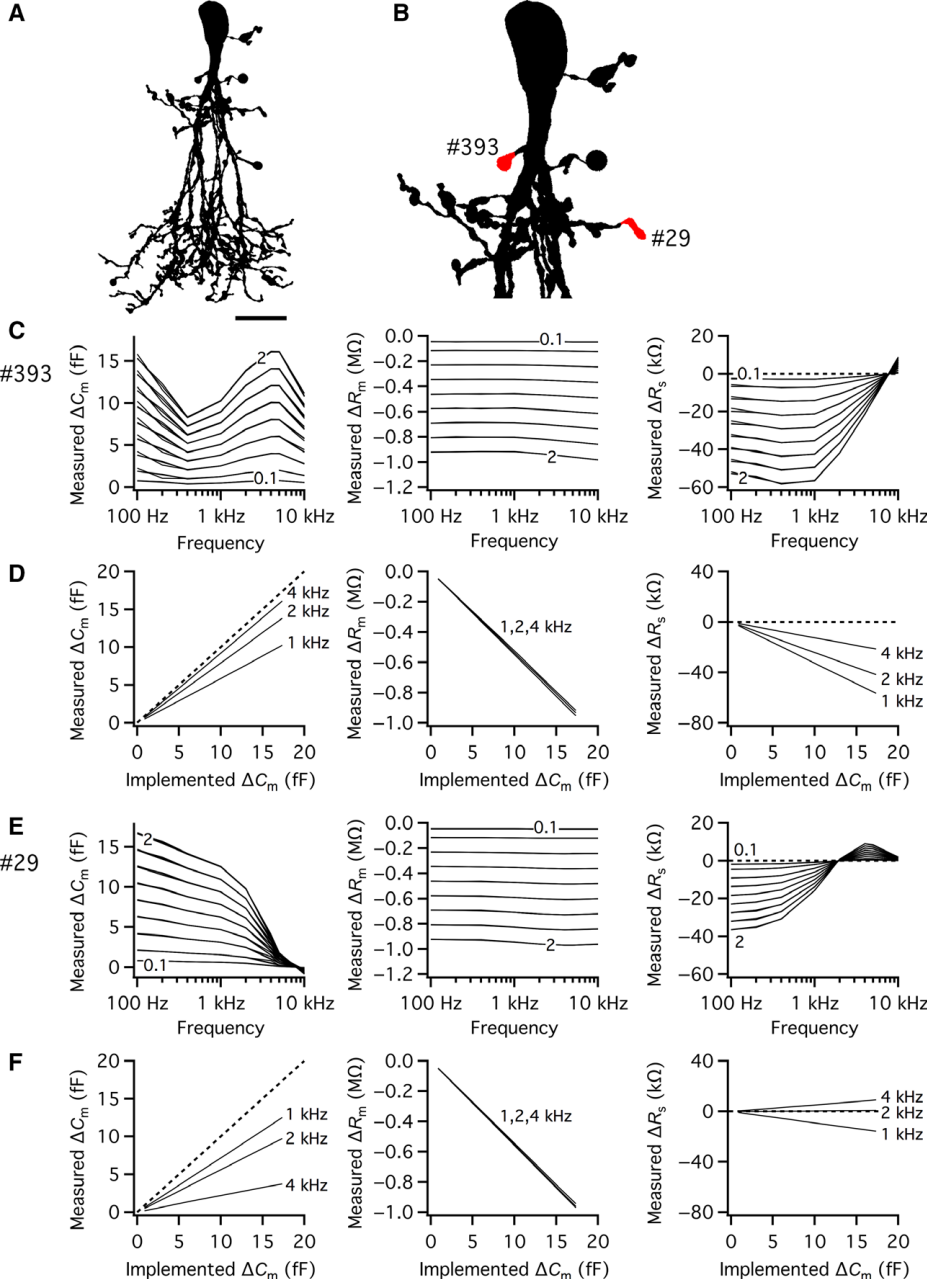
Accuracy of *ΔC*_m_ estimates as a function of magnitude and location of capacitance increase at AII amacrine lobular appendages. (A) Shape plot of morphologically reconstructed AII amacrine cell used for computer simulations. (B) Enlarged shape plot of same AII cell restricted to region with soma and lobular dendrites and appendages. (C) Measured changes in capacitance (*ΔC*_m_; left), membrane resistance (*ΔR*_m_; middle), and series resistance (*ΔR*_s_; right) as a function of sine wave stimulus frequency (100 Hz–10 kHz) after increasing the membrane capacitance of a specific lobular appendage (indicated by section number to the left) by 0.868–17.36 fF, corresponding to an increase of surface area of 0.1–2 *µ*m^2^ (in steps of 0.25 *µ*m^2^) with specific capacitance 0.868 *µ*F/cm^2^. Each trace corresponds to a specific increase of surface area, increasing from 0.1 to 2 *µ*m^2^ (as indicated). Here and in (E) each condition was simulated for three different values of *R*_s_ (*R*_s(theory)_; 1, 50, and 250 M*Ω*). (D) Relationship between magnitude of implemented and measured *ΔC*_m_ (left), *ΔR*_m_ (middle), and *ΔR*_s_ (right) for three different values of sine wave stimulus frequency (1, 2, and 4 kHz; as indicated). Here and in (F) data are shown for one value of *R*_s_ (1 M*Ω*) and the identity line (broken line) in left panel corresponds to identical values of implemented and measured values of *ΔC*_m_. Note the approximately linear relationship, but with different slope, between implemented and measured values of *ΔC*_m_ for the examined sine wave stimulation frequencies (left). Lobular appendage and data as in (C). (E) Measured changes in capacitance (*ΔC*_m_; left), membrane resistance (*ΔR*_m_; middle), and series resistance (*ΔR*_s_; right) as a function of sine wave stimulus frequency (100 Hz–10 kHz) after increasing the membrane capacitance of a specific lobular appendage (indicated by section number to the left) by 0.868–17.36 fF (as in *C*). (F) Relationship between magnitude of implemented and measured *ΔC*_m_ (left), *ΔR*_m_ (middle), and *ΔR*_s_ (right) for three different values of sine wave stimulation frequency (1, 2, and 4 kHz; as indicated). Note the approximately linear relationship, but with different slope, between implemented and measured values of *ΔC*_m_ for the examined sine wave stimulation frequencies (left). Lobular appendage and data as in (E). Scale bar: 10 *µ*m (A).

It is of considerable interest to understand the degree of linearity between the magnitude of the true (theoretical) increase of capacitance at a specific lobular appendage and the magnitude of the estimated capacitance increase. To explore this quantitatively, we performed simulations where the capacitance increase for a given lobular appendage was varied by increasing the membrane surface area between 0 and 2 *µ*m^2^, with steps of 0.25 *µ*m^2^ and starting at 0.1 *µ*m^2^. This corresponds to 0 to ~20 fF added to each lobular appendage, depending on the exact value of the specific membrane capacitance for each cell. For each lobular appendage, the sine wave frequency ranged from 100 Hz–10 kHz. Each simulation condition was repeated for *R*_s_ values of 1, 50, and 250 M*Ω*. For the AII amacrine cell illustrated in Figure 5A, we examined five different lobular appendages and the results for two of them (Fig. 5B) are illustrated in Figure 5C–F. The first lobular appendage (section #393) was located relatively close to the soma and was directly connected to the apical dendrite via a short and relatively thick dendrite. The second lobular appendage (section #29) was located further from the soma and indirectly connected to the distal part of the apical dendrite via a series of branching processes. The results for *ΔC*_m_, *ΔR*_m_, and *ΔR*_s_ are illustrated in Figure 5C and D for the proximal (#393) lobular appendage and in Fig. 5E and F for the distal (#29) lobular appendage. It is immediately apparent that the value of *R*_s_ has essentially no effect on the estimates, as the three conditions were almost completely superimposed in all graph panels.

For the proximal lobular appendage (#393), the estimates of *ΔC*_m_ seemed relatively linear over a relatively large range of frequencies, up to ~10 kHz, although the accuracy of the estimates depended strongly on the sine wave frequency (Fig. 5C; left panel). The degree of linearity was examined in more detail by plotting the estimated *ΔC*_m_ values for 1, 2, and 4 kHz versus the magnitude of the implemented *ΔC*_m_ (Fig. 5D; left panel). For all three sine wave frequencies, the relation between measured and implemented *ΔC*_m_ was linear, but the slope varied and only approached a value of 1 (corresponding to the identity line) for 4 kHz. The relation between the implemented *ΔC*_m_ and the measured *ΔR*_m_ was essentially linear for all sine wave frequencies, with only negligible influence of the sine wave frequency (Fig. 5C and D; middle panels) and was explained by the expected reduction of *R*_m_ for the increased membrane surface area. For *R*_s_, the relationship between sine wave frequency, implemented *ΔC*_m_ value and measured *ΔR*_s_ value was more complicated, with increasing understimation of *R*_s_ at low sine wave frequencies (Fig. 5C and D; right panels), a cross‐over point at a frequency of ~6–7 kHz, and slight overestimation of *R*_s_ up to a frequency of 10 kHz (Fig. 5C; right panel). Qualitatively similar results were seen for other lobular appendages located relatively close to the soma.

For the more distally located lobular appendage (#29), the results were qualitatively similar, but differed with respect to some important details. For sine wave frequencies of 1, 2, and 4 kHz, the relation between measured and implemented *ΔC*_m_ was relatively linear (Fig. 5E and F; left panels), but with strong attenuation for the latter frequency. As the accuracy dropped monotonically for increasing sine wave frequencies (Fig. 5E; left panel), the slope was higher for 1 than for 2 kHz (Fig. 5F; left panel). The relation between the implemented *ΔC*_m_ and the measured *ΔR*_m_ was almost identical to that observed for the more proximally located lobular appendage, indicating that the changes of *R*_m_ are independent of the location of the capacitance increase (Fig. 5C and D vs. Fig. 5E and F; middle panels). For estimates of *ΔR*_s_, the specific sine wave frequency determined whether the increase of *ΔC*_m_ appeared together with an increase (4 kHz), a decrease (1 kHz) or almost no change of *R*_s_ (2 kHz; Fig. 5E and F; right panels). These results suggest a strong limitation of quantitatively resolving changes in capacitance at more distally located lobular appendages when using sine wave frequencies >1 kHz.

### Detecting capacitance increases occurring in lobular appendages located at different anatomical distances from the soma

**Figure 6:**
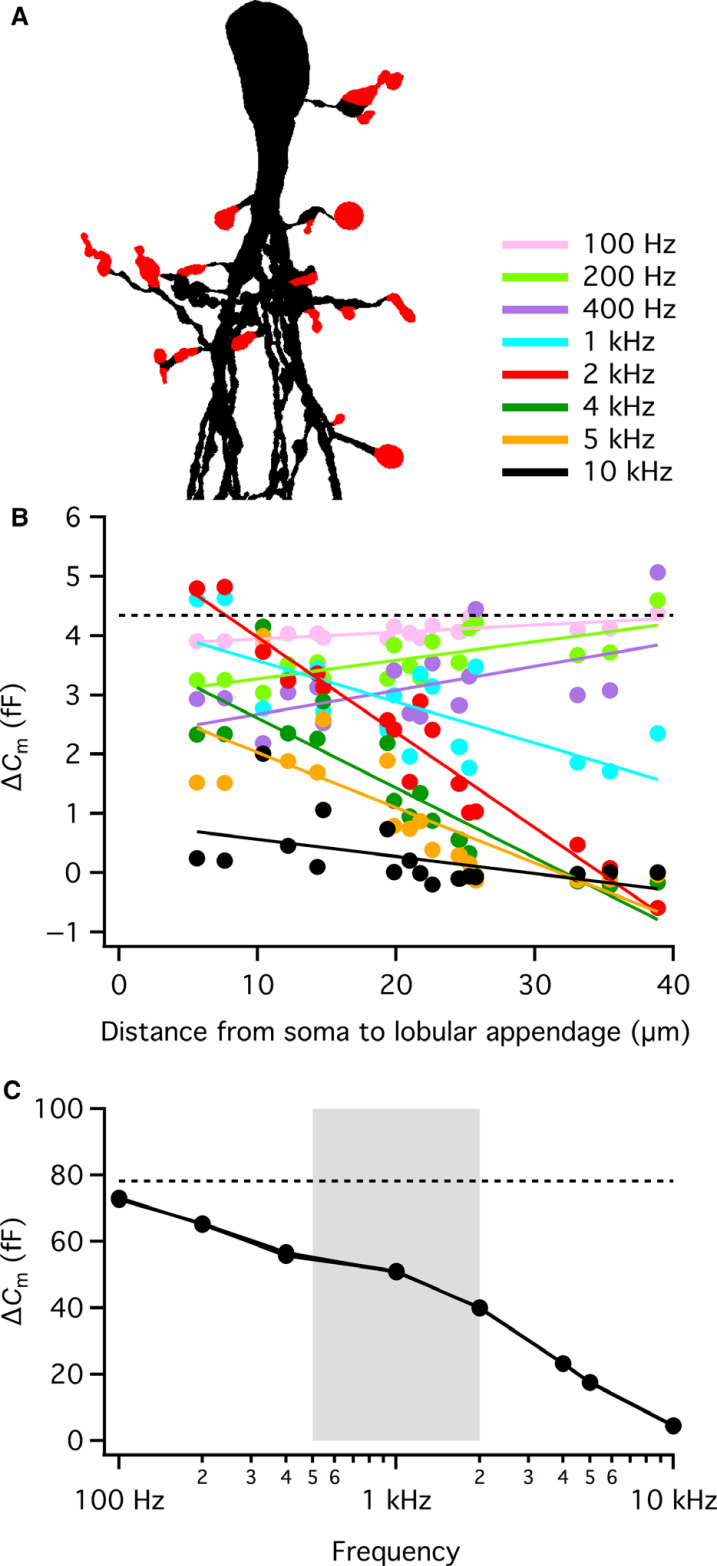
Accuracy of *ΔC*_m_ estimates as a function of distance of capacitance increase at lobular appendages from AII amacrine soma. (A) Shape plot of morphologically reconstructed AII amacrine cell used for computer simulations and the lobular appendages where an increase of capacitance was simulated (red). (B) Relationship between anatomical (non‐Euclidean) distance of lobular appendage with increased capacitance from soma and magnitude of measured change in capacitance (*ΔC*_m_) as a function of sine wave stimulation frequency (100 Hz–10 kHz). For each lobular appendage, the membrane capacitance was increased by 4.34 fF (corresponding to an increase of surface area of 0.5 *µ*m^2^ with specific capacitance 0.868 *µ*F/cm^2^). For a given sine wave stimulation frequency, the results for all lobular appendages (*n* = 18) are displayed by filled circles of the same color (see figure for color code) and the data points have been fitted with a straight line (same color). Note that for the lowest sine wave frequencies, the accuracy of *ΔC*_m_ measurements do not depend on the distance of the lobular appendage from the soma. With increasing frequency, the accuracy drops with increasing distance, but for the highest frequency, the accuracy is low and almost independent of distance. (C) Measured changes in capacitance (*ΔC*_m_) as a function of sine wave stimulus frequency (100 Hz–10 kHz) after simultaneously increasing the membrane capacitance of each of the 18 lobular appendages (as in B), corresponding to a total increase of surface area of 9 *µ*m^2^ (~78 fF). Each condition was simulated for three different values of *R*_s_ (*R*_s(theory)_; 1, 50, and 250 M*Ω*). Note the marked influence of sine wave frequency on accuracy of estimate of *ΔC*_m_. The shaded rectangle (gray) marks the range of sine wave frequencies for which capacitance measurements were explored with physiological recordings of mouse AII amacrine cells by Balakrishnan et al. (2015). Scale bar: 10 *µ*m (A).

To further investigate the distance‐dependence of exocytosis measurements, we systematically examined the relationship between the measured value of *ΔC*_m_ (for a constant value of implemented *ΔC*_m_) and the anatomical (non‐Euclidean) distance of the corresponding lobular appendage from the soma. For the AII amacrine cell illustrated in Figure 6A, an increase of surface area was simulated individually for 18 different lobular appendages (0.5 *µ*m^2^, corresponding to *ΔC*_m(theory)_ = 4.34 fF; Fig. 6B, broken line). The anatomical distance was measured as the distance from the center of the soma (the location of the recording electrode) to the center of the lobular appendage along the dendritic tree. The distance from the soma to each of the lobular appendages ranged from ~6 to ~39 *µ*m (Fig. 6B). In each case, the sine wave frequency ranged from 100 Hz–10 kHz. As illustrated in Figure 6B, for the lowest sine wave frequencies (100–400 Hz) there was little influence of the distance and the accuracy was relatively high for both 100 and 200 Hz. For higher sine wave frequencies (1–5 kHz), however, there was a clear influence of distance, such that the measured value of *ΔC*_m_ decreased with increasing distance of the site of capacitance increase from the recording electrode at the soma (Fig. 6B). Overall, for any given distance there was also a reduction in accuracy with increasing sine wave frequency (Fig. 6B). For 10 kHz (the highest frequency examined), there was essentially no difference between different locations, as only one lobular appendage gave rise to a detectable *ΔC*_m_ (Fig. 6B).

The results presented for individual lobular appendages in Figures 4, 5, and 6, suggest that overall, measurements with high accuracy will be obtained up to a certain sine wave frequency, beyond which the accuracy will drop. The frequency that results in the highest accuracy will be reduced as the distance from the soma to a given lobular appendage increases. We next asked how the sine wave frequency will influence the accuracy in the experimentally more relevant situation, when the capacitance increase is distributed across multiple lobular appendages. To investigate this, we simulated an increase of surface area simultaneously for the same 18 lobular appendages illustrated in Figure 6A and B (0.5 *µ*m^2^), corresponding to a total increase of 9 *µ*m^2^ (~78 fF). The results displayed in Figure 6C indicated that the accuracy dropped gradually from almost 1 at 100 Hz to almost zero at 10 kHz. Importantly, when we plotted the corresponding function obtained by algebraically adding the results obtained for independent measurements at the same 18 lobular appendages, the curves overlapped completely (data not shown). For the frequency range (500 Hz–2 kHz; Fig. 6C) explored in the physiological recordings by Balakrishnan et al. (2015), the difference in accuracy was relatively small, thus it is unlikely to be detected in the presence of noise and variability in physiological recordings. However, while there might be little difference in accuracy for sine wave frequencies in this range, the overall accuracy is low (~50% in this example) and suggests that physiological measurements with these frequencies will significantly underestimate the true size of the release pool(s).

### Influence of R_m_ and R_i_ on the performance of the “Sine + DC” technique for measuring baseline capacitance and capacitance increases

For our compartmental models of AII amacrine cells, the average *R*_m_ was 30.2 k*Ω* cm^2^ and the average *R*_i_ was 198 *Ω* cm (Zandt et al. 2018). Both estimates were obtained at room temperature and were associated with a certain degree of variability and error (for quantitative analysis, see Zandt et al. 2018). In addition, similar analyses of different neurons in the CNS have provided estimates for the passive membrane properties that display a range of values. Physiological experiments with capacitance measurement of exocytosis during whole‐cell patch‐clamp recording are in general performed with a Cs^+^‐based intracellular solution. This facilitates measuring the net amplitude of voltage‐gated Ca^2+^ current by blocking K^+^ currents which also increases *R*_m_. In addition, if physiological recordings are performed at higher (more physiological) temperatures, *R*_i_ will be lower (Trevelyan and Jack 2002). On this background, we decided to explore the influence of both *R*_m_ and *R*_i_ on the performance of the “Sine + DC” technique for measuring both baseline capacitance and changes in capacitance reflecting exocytosis in AII amacrine cells.

**Figure 7:**
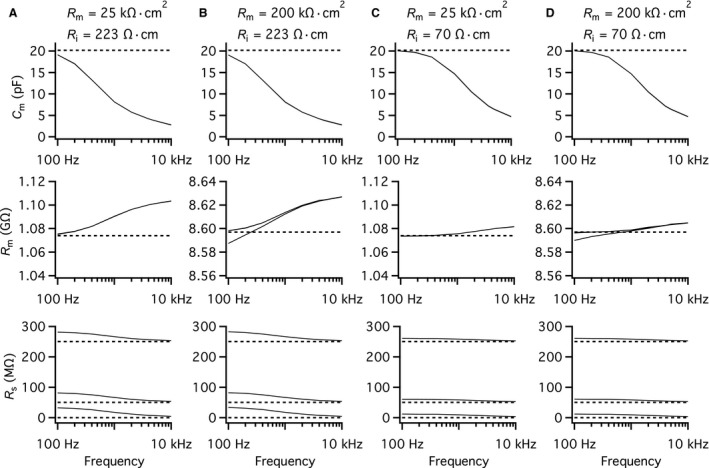
Influence of the passive membrane (*R*_m_) and cytoplasm (*R*_i_) properties on the performance of the “Sine + DC” technique for measuring baseline capacitance of AII amacrine cells. The AII amacrine cell used for the computer simulations is the same as illustrated in Figure 6. (A–D) Estimates of total capacitance (*C*_m_; top row), total membrane resistance (*R*_m_; middle row), and series resistance (*R*_s_; bottom row) as a function of sine wave stimulation frequency (100 Hz–10 kHz) for different values of specific membrane resistance (*R*_m_) and cytoplasmic resistivity (*R*_i_). For each condition, the simulations were repeated for three different values of *R*_s_ (*R*_s(theory)_; 1, 50, and 250 M*Ω*). In (A) *R*_m_ and *R*_i_ were set to the original values estimated by compartmental model fitting (Zandt et al. 2018). In (B) *R*_m_ was increased to 200 k*Ω* cm^2^ while *R*_i_ was kept at its original value. In (C) *R*_i_ was decreased to 70 *Ω* cm while *R*_m_ was kept at its original value. In (D) *R*_m_ was increased to 200 k*Ω* cm^2^, while *R*_i_ was decreased to 70 *Ω* cm. Theoretical parameter values: *C*_m_ = 20.19 pF (A–D); *R*_m_ = 1.744 G*Ω* (A and C), 8.597 G*Ω* (B and D).

We modified the values for *R*_m_ and *R*_i_ to obtain different conditions with minimum and maximum dendritic attenuation, following the approach of Spruston et al. (1993) in their modeling study of electrotonic signaling in hippocampal CA3 pyramidal neurons. The minimum attenuation condition was modeled with high *R*_m_ (200 k*Ω* cm^2^), to reflect the use of channel blockers (including Cs^+^ to block K^+^ channels), and low *R*_i_ (70 *Ω* cm), with the latter parameter value taken from estimates in cortical pyramidal neurons (Barrett and Crill 1974). We repeated the “Sine + DC” capacitance simulations for one of the AII amacrine compartmental models (same cell as in Figs. 5 and 6) for three different conditions in addition to the condition with the original values of *R*_m_ and *R*_i_ (the three panels of Fig. 7A show the baseline estimates of *C*_m_, *R*_m_ and *R*_s_ as a function of sine wave frequency). First, *R*_m_ was increased to 200 k*Ω* cm^2^ (and *R*_i_ kept at the original value of 223 *Ω* cm; Fig. 7B). Second, *R*_i_ was decreased to 70 *Ω* cm (and *R*_m_ kept at the original value of 25 k*Ω* cm^2^; Fig. 7C). Finally, *R*_m_ was increased to 200 k*Ω* cm^2^ and *R*_i_ was decreased to 70 *Ω *cm (Fig. 7D). The increase of *R*_m_ had virtually no effect on the baseline estimates of *C*_m_, *R*_m_ or *R*_s_ (Fig. 7B). In contrast, when *R*_i_ was reduced, irrespective of whether it was combined with the original (Fig. 7C) or the increased value (Fig. 7D) of *R*_m_, it had a marked influence on the baseline estimates. First, the measured value of *C*_m_ increased for all sine wave frequencies, although the estimates still decreased with increasing frequency (Fig. 7C and D; upper row). Second, the overestimation of *R*_m_ at medium and higher frequencies was reduced (Fig. 7C and D; middle row). Finally, the overestimation of *R*_s_ at the lower frequencies was reduced (Fig. 7C and D; bottom row). In conclusion, a reduction of *R*_i_ increased the overall accuracy and reduced the influence of the differences in sine wave frequency.

**Figure 8:**
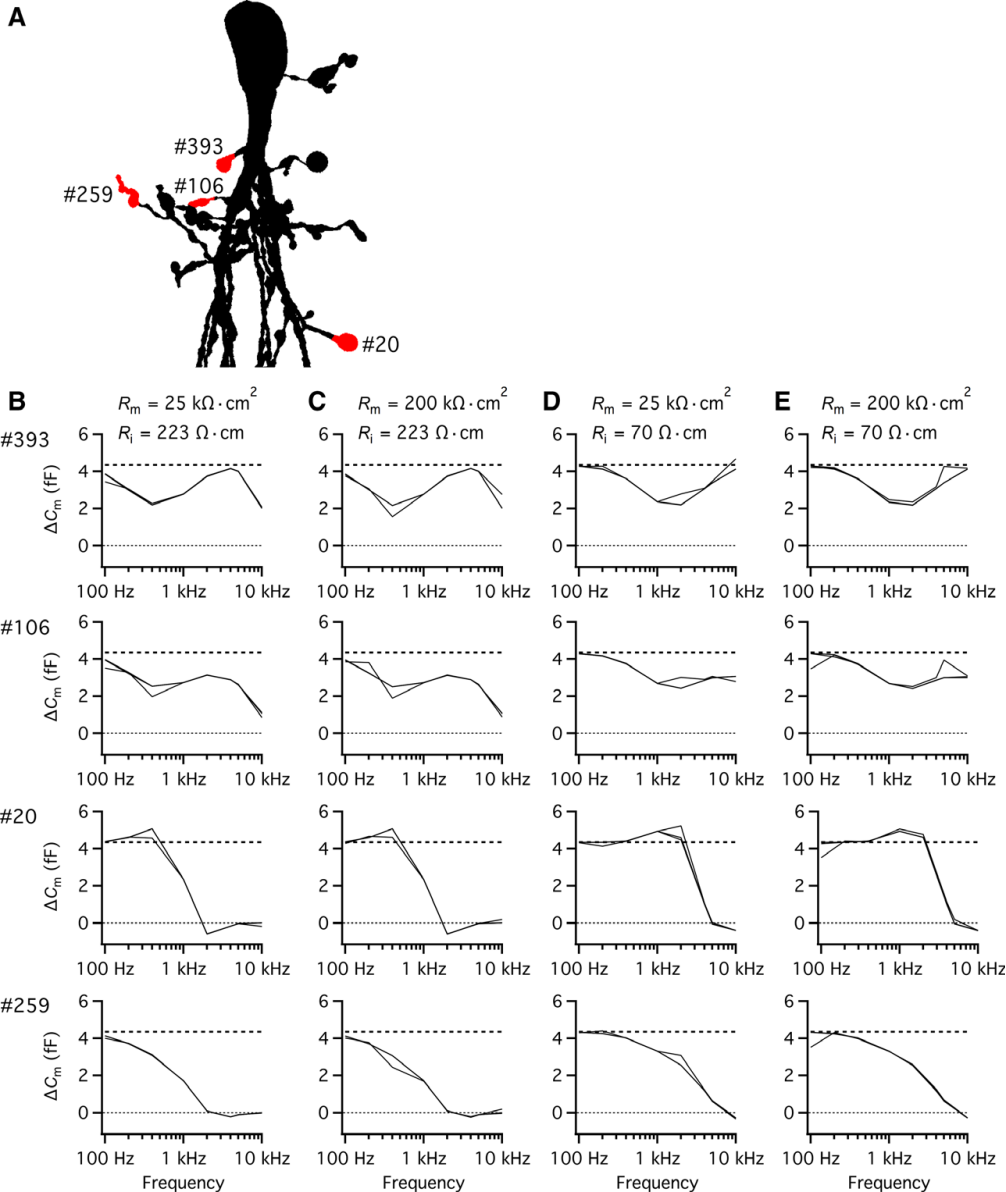
Influence of the passive membrane (*R*_m_) and cytoplasm (*R*_i_) properties on the performance of the “Sine + DC” technique for measuring capacitance increase at different lobular appendages of an AII amacrine cell with computer simulations of soma recording. (A) Enlarged shape plot (restricted to region with soma and lobular dendrites and appendages) of morphologically reconstructed AII amacrine cell used for computer simulations. (B–E) Estimates of changes in capacitance (*ΔC*_m_) as a function of sine wave stimulation frequency (100 Hz–10 kHz) after increasing the membrane capacitance of a lobular appendage by 4.34 fF (corresponding to an increase of surface area of 0.5 *µ*m^2^ with specific capacitance 0.868 *µ*F/cm^2^). Each column displays the results for a given combination of values for *R*_m_ and *R*_i_ (as indicated). The rows correspond to different lobular appendages (indicated by section number to the left) and are arranged in order of increasing anatomical and electrotonic distance from soma. For each condition, the simulations were repeated for three different values of *R*_s_ (*R*_s(theory)_; 1, 50, and 250 M*Ω*).

We next examined how the modified values of *R*_m_ and *R*_i_ might influence the ability of the “Sine + DC” technique to detect localized increases of membrane surface area and thereby *ΔC*_m_. We simulated increased surface area (0.5 *µ*m^2^) and capacitance at the same lobular appendages (Fig. 8A) as were used in the condition with the original values for *R*_m_ and *R*_i_ and for each case the results were compared to those obtained for the unmodified morphology. The results for four lobular appendages are illustrated in Figure 8B–E. Increasing *R*_m_ to 200 k*Ω* cm^2^, in the absence of a change in *R*_i_ had no effect on the accuracy of *ΔC*_m_ estimates for any of the lobular appendages examined (compare Fig. 8B and C). In contrast, however, reducing *R*_i_ to 70 *Ω* cm increased the accuracy of *ΔC*_m_ estimates for most lobular appendages, depending on the distance of the lobular from the soma, with the most marked effect observed for medium and higher frequencies (compare Fig. 8B and D). Qualitatively, decreasing *R*_i_ resulted in a rightward shift of the *ΔC*_m_ versus sine wave frequency functions. The effect of reduced *R*_i_ was the same, irrespective of whether it was changed alone (Fig. 8D) or simultaneously with an increase of *R*_m_ (Fig. 8E). Both *ΔR*_m_ and *ΔR*_s_ were determined with high accuracy for the different conditions with increased *R*_m_ and/or reduced *R*_i_ (data not shown).

### Influence of gap junction coupling on performance of the “Sine + DC” technique for measuring baseline capacitance and capacitance increases

**Figure 9:**
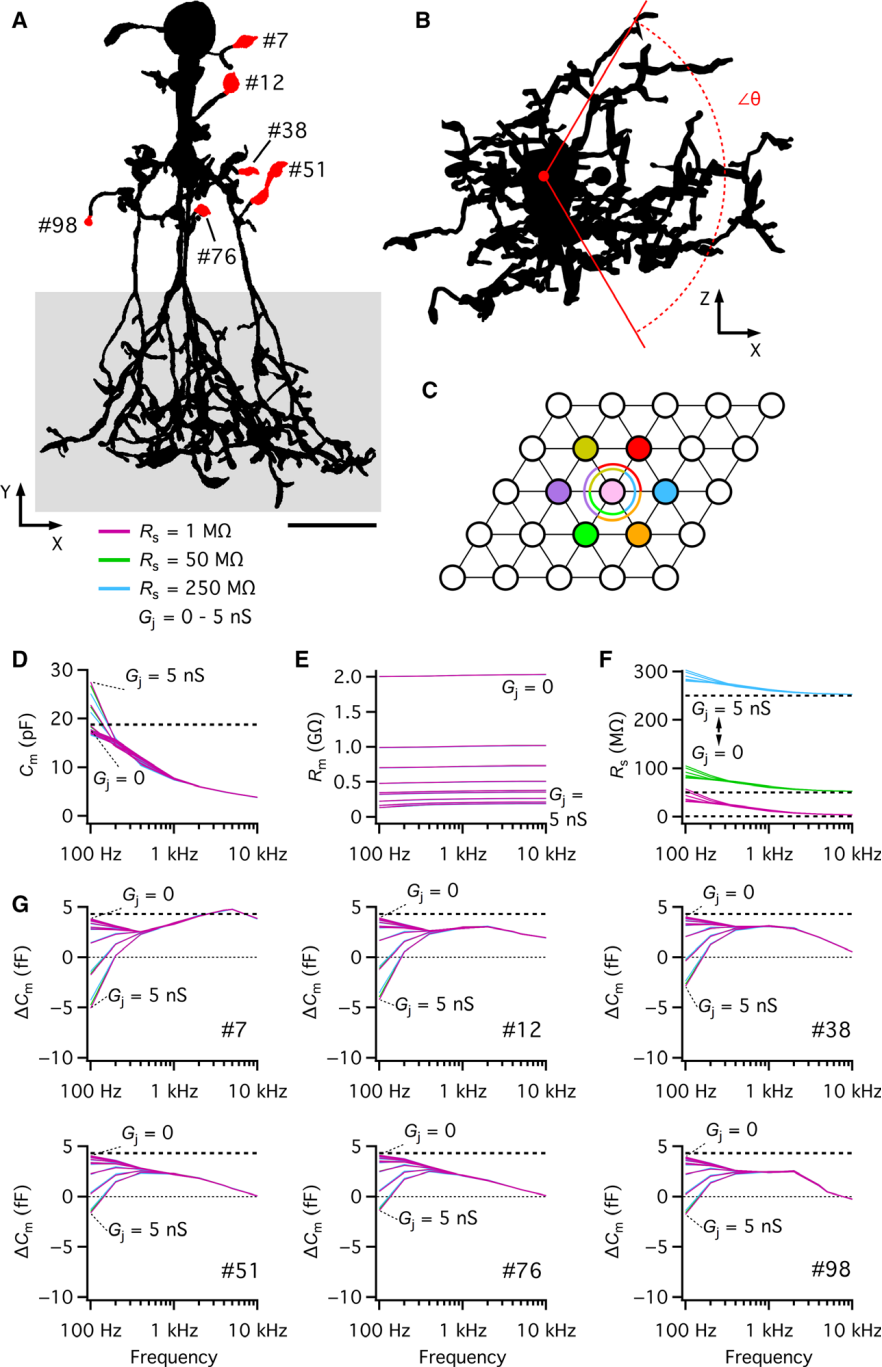
Influence of gap junction coupling and junctional conductance (*G*_j_) on performance of the “Sine + DC” technique for measuring baseline capacitance and capacitance increase at different lobular appendages of an AII amacrine cell with computer simulations of soma recording. (A) Shape plot of morphologically reconstructed AII amacrine cell used for computer simulations, viewed from the front (along the *Z* axis defined during the MPE microscopic imaging). The shaded region indicates the height (range along the *Y* axis) where contacts via electrical synapses could be made with the cell's nearest neighbors (corresponding to S5, S4, and the proximal part of S3 in the inner plexiform layer). (B) Shape plot of same AII amacrine cell, viewed from the bottom (along the *X* axis). The red circle corresponds to the projected location of the center of the soma. The displayed angle *θ* (=120°) corresponds to the sector within which the cell would make contacts via electrical synapses with one of its nearest neighbors (corresponding to the cell labeled by the filled blue circle in C). (C) Hexagonal network of 25 electrically coupled AII amacrine cells arranged in a trapezoidal grid (5 × 5) used for computer simulations. Each circle corresponds to a cell and each black line corresponds to electrical coupling between two cells (mediated by gap junctions). The sectors within which gap junctions were made between the center cell (pink) and any of its six nearest neighbor cells are indicated by a 120° arc of the same color as the corresponding neighbor. Stimuli were applied to center cell. (D–F) Influence of *G*_j_ ranging from 0 to 5 nS (0, 100, 200, 400, 700, 800, 1600, 3200 and 5000 pS) on estimates of total capacitance (*C*_m_; D), total membrane resistance (*R*_m_; E), and series resistance (*R*_s_; F) as a function of sine wave stimulation frequency (100 Hz–10 kHz). The broken horizontal line in (D) indicates the capacitance (18.74 pF) of the isolated cell (A). For each condition, the simulations were repeated for three different values of *R*_s_ (*R*_s(theory)_; 1, 50, and 250 M*Ω*; here and in (G) color code displayed above panel D). (G) Influence of *G*_j_ (0–5 nS) on estimates of changes in capacitance (*ΔC*_m_) as a function of sine wave stimulation frequency (100 Hz–10 kHz) after increasing the membrane capacitance of a lobular appendage (indicated by section number in each panel) of the AII amacrine in the center of the network by 4.32 fF (corresponding to an increase of surface area of 0.5 *µ*m^2^ with specific capacitance 0.864 *µ*F/cm^2^). For each condition, the simulations were repeated for three different values of *R*_s_ (*R*_s(theory)_; 1, 50, and 250 M*Ω*). Scale bar: 10 *µ*m (A).

It is well established that AII amacrine cells are electrically coupled to other AII amacrine cells as well as to ON‐cone bipolar cells (Veruki and Hartveit 2002a,2002b), with both sets of connections primarily made at the arboreal dendrites in the inner plexiform layer. For rat retina, ultrastructural investigations have found the heterologous (AII–ON‐cone bipolar) gap junctions in S3 and S4 and the homologous (AII–AII) gap junctions in S5 (Chun et al. 1993). The compartmental models of AII amacrine cells used in the simulations described above were developed for cells where gap junction coupling had been blocked pharmacologically with MFA (Zandt et al. 2018). To construct computational models of gap junction‐coupled cells, we arranged identical copies of an AII compartmental model (Fig. 9A and B) in a hexagonal network (Fig. 9C), with 20 individual gap junction contacts located in a specific sector of the XZ plane (Fig. 9B) for each pairwise intercellular connection (see Methods). With this network, each AII amacrine cell is electrically coupled to six nearest neighbors (cf. Wässle et al. 1993; Veruki et al. 2008). The simulated network of electrically coupled AII amacrine cells did not incorporate ON‐cone bipolar cells explicitly, but to (partially) compensate for this, we allowed contacts between neighboring AII amacrine cells to be made across S3 (the proximal part), S4 and S5 of the inner plexiform layer (see Methods). We assume that this did not change the properties of the electrical network in any fundamental way. All simulations reported here were run with a network of 25 cells arranged in a 5 × 5 trapezoidal grid (Fig. 9C). For each condition with increased surface area of a specific lobular appendage of the AII amacrine located in the center of the network (Fig. 9A and C), we varied the junctional conductance homogeneously for all pairwise connections between 0 pS (corresponding to an uncoupled network) and 5000 pS.

We first investigated how varying the junctional conductance influenced the baseline capacitance measurements. Qualitatively, we expected that because of frequency‐dependent passive attenuation in AII amacrine cells, low‐frequency sine wave voltage stimuli (applied at the soma of the center cell; Fig. 9C) will “see” a larger fraction of the capacitance of the coupled cells than high‐frequency stimuli. For the highest sine wave frequencies, we expected the capacitance estimates to be unaffected by electrical coupling because the voltage fluctuations would be too strongly attenuated through the dendritic tree, with little or no transmission across the gap junctions (Veruki and Hartveit 2002a,2002b ; Zandt et al. 2018). For the gap junction‐coupled network based on the AII amacrine cell illustrated in Figure 9A, we simulated capacitance measurements applied to the center cell with pairwise junctional conductance between 100 and 5000 pS and sine wave frequency between 100 Hz and 10 kHz. As for earlier simulations, *R*_s_ was set to 1, 50 or 250 M*Ω*. Overall, *R*_s_ had only a minor influence on the estimated capacitance and the curves overlap. At the highest sine wave frequencies (≥2 kHz), there was no influence of gap junction coupling on the estimates of *C*_m_ (Fig. 9D). For sine wave frequencies between 200 Hz and 1 kHz, differences in junctional conductance had a small (~1 pF), but noticeable effect on the estimated capacitance (Fig. 9D). In contrast, for a sine wave frequency of 100 Hz, *C*_m_ was strongly overestimated for the two highest values of junctional conductance (3200 and 5000 pS; Fig. 9D). For junctional conductances between 100 and 1600 pS, however, the *C*_m_ estimates only differed by ~1 pF (Fig. 9D). These results suggest that overall, “Sine + DC” lock‐in estimates of the capacitance of AII amacrine cells are remarkably unaffected by electrical coupling between the cells. Compared to the uncoupled state, a clear difference is only seen for the combination of low sine wave frequency and high junctional conductance. The estimates of *R*_m_ were strongly influenced by the strength of electrical coupling, with decreasing values of *R*_m_ for increasing values of junctional conductance (Fig. 9E). For *R*_s_, there was essentially no influence of the strength of coupling for sine wave frequencies ≥400 Hz (Fig. 9F). These results are very similar to the corresponding results obtained with physiological recording (Fig. 3B–G).

In the next series of simulations, we increased the surface area of 12 different lobular appendages in turn (each by 0.5 *µ*m^2^) for the center AII amacrine (Fig. 9C) and repeated the “Sine + DC” capacitance measurements for each case. The results for six of these lobular appendages (Fig. 9A) are illustrated in Figure 9G and demonstrate that for sine wave frequencies ≥ 400 Hz, there was almost no discernible effect of electrical coupling on the accuracy of *ΔC*_m_ estimates compared to the uncoupled condition. In contrast, for sine wave frequencies < 400 Hz, electrical coupling had a marked effect on the accuracy of *ΔC*_m_ estimates, with greatly reduced accuracy for the higher values of junctional conductance. Indeed, for the two highest conductance values (3200 and 5000 pS), *ΔC*_m_ was ~0 or negative for all lobular appendages examined (Fig. 9G). For the lowest values of junctional conductance, the influence of coupling on the accuracy of *ΔC*_m_ estimates was negligible (Fig. 9G). Taken together, these results suggest that “Sine + DC” measurements of *ΔC*_m_ are resistant to the effects of electrical coupling when the sine wave frequency is higher than 1 kHz. For frequencies lower than 1 kHz, the magnitude of *ΔC*_m_ can be markedly underestimated, but only for high values of junctional conductance. Unfortunately, this means that the potential advantage of a low sine wave frequency (~100 Hz) for measuring *ΔC*_m_ with high accuracy for exocytosis at AII lobular appendages, independently of their location in the dendritic tree, is most likely compromised in the presence of moderate to strong electrical coupling.

### Performance of dendritic recording to detect capacitance increase occurring at a lobular appendage

**Figure 10:**
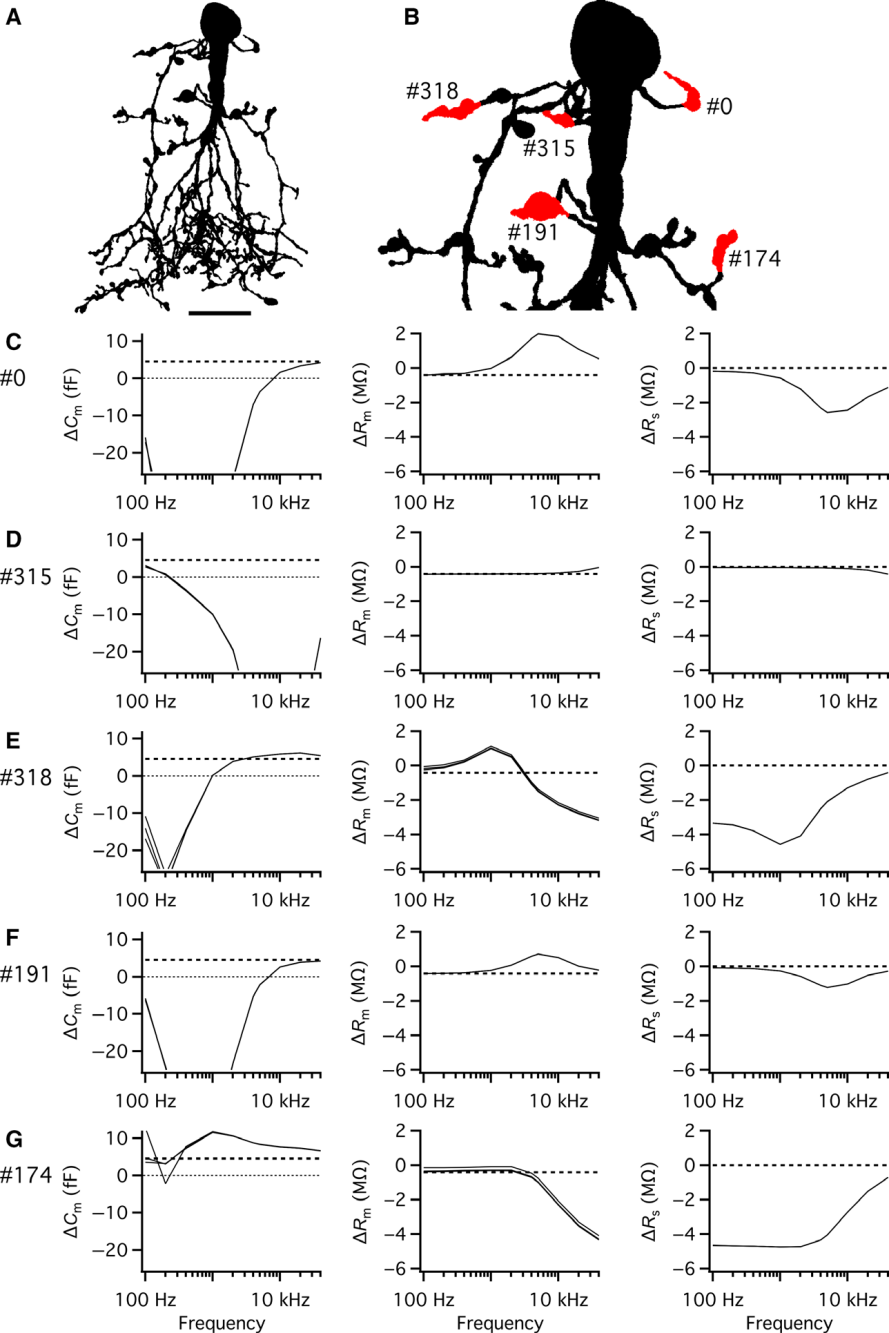
Performance of dendritic recording and the “Sine + DC” technique for measuring capacitance increase at individual lobular appendages of an AII amacrine cell. (A) Shape plot of morphologically reconstructed AII amacrine cell used for computer simulations. (B) Enlarged shape plot of same AII cell restricted to region with soma and lobular dendrites and appendages. (C–G) Estimates of changes in capacitance (*ΔC*_m_; left column), membrane resistance (*ΔR*_m_; middle column), and series resistance (*ΔR*_s_; right column) as a function of sine wave stimulation frequency (100 Hz–10 kHz) after increasing the membrane capacitance of a lobular appendage (indicated by section number to the left) by 4.51 fF (corresponding to an increase of surface area of 0.5 *µ*m^2^ with specific capacitance 0.9016 *µ*F/cm^2^). The theoretical value of *ΔR*_m_ was −0.42 M*Ω*. The rows (corresponding to different lobular appendages) are arranged approximately in order of increasing anatomical and electrotonic distance from soma. For each condition, the simulations were repeated for three different values of *R*_s_ (*R*_s(theory)_; 1, 50, and 250 M*Ω*). For improved display of the functionally most important results, several of the *ΔC*_m_ versus frequency functions do not display the full range along the *Y* axis (C–G; left column). Scale bar: 10 *µ*m (A).

Our computational modeling of capacitance measurements with the recording electrode located at the cell body suggests that exocytosis cannot be measured with high accuracy for all lobular appendages of an AII amacrine cell, as they are located at different anatomical and electrotonic distances from the cell body. In principle, the problem can be solved be reducing the sine wave frequency, but a generally high accuracy is only reached when the frequency becomes so low (e.g. 100 Hz) that the measurements are influenced by the electrical coupling of AII amacrine cells. On this background, it is worth considering if direct recording from a lobular appendage might allow estimating the capacitance increase following exocytosis from the same lobular appendage with high accuracy, independently of location. The size of some of the larger lobular appendages is commensurate with the size of processes and terminals in other neurons where such recordings have been successfully obtained (Hsu and Jackson 1996; Hallermann et al. 2003; Oltedal and Hartveit 2010). Qualitatively, detecting the increase of capacitance occurring at a lobular appendage will likely require high sine wave frequencies such that the signal is confined as much as possible to the local region where the recording pipette is positioned. In anticipation of such potential physiological recordings, we performed simulations of capacitance recordings with the electrode located at individual lobular appendages. In all cases, the measured value of (baseline) *C*_m_ decayed more rapidly with increasing sine wave frequency compared to simulations with a somatic electrode (data not shown), as is expected from the stronger attenuation for signals generated in the dendritic tree than at the soma (Zandt et al. 2018). To investigate the ability of lobular recordings to measure local capacitance increases with high accuracy, we simulated an increase of local surface area by 0.5 *µ*m^2^. For the AII amacrine cell illustrated in Figure 10A, the increase in surface area corresponded to a nominal capacitance increase of 4.51 fF. For each condition, the simulations were repeated for three different values of *R*_s_ (*R*_s(theory)_; 1, 50, and 250 M*Ω*).

The results for five lobular appendages (Fig. 10B), located at different distances from the cell body, are illustrated in Figure 10C–G. For the majority of lobular appendages examined in this way (in this and another cell), a sine wave frequency of 100 Hz resulted in a negative *ΔC*_m_ estimate (Fig. 10C, E, and F; left panels). With increasing sine wave frequency, *ΔC*_m_ typically increased in the negative direction (Fig. 10C–G) to ~−100 fF (data not shown). With further increase of the sine wave frequency, the magnitude of the negative *ΔC*_m_ decreased and typically reached a cross‐over point, corresponding to a change from negative to positive *ΔC*_m_ values (Fig. 10C, E–G). To examine this over a larger range of sine wave frequencies, the highest frequency was extended from 10 to 40 kHz for all lobular appendages. For some lobular appendages, however, the cross‐over point was not reached even at 40 kHz (Fig. 10D) and had to be increased even further (to 80 and 160 kHz) to result in a positive *ΔC*_m_ and high accuracy (data not shown). For some lobulars we observed that high sine wave frequencies could lead to moderate overestimation of *ΔC*_m_ (Fig. 10E and G).

For the estimates of *ΔR*_m_ and *ΔR*_s_, the results varied between different lobular appendages. *ΔR*_m_ could be both over‐ and under‐estimated relative to the expected decrease and for some lobulars this depended on the sine wave frequency (Fig. 10C–G; middle panels). In addition, a local increase of surface area evoked an apparent decrease of *R*_s_ (Fig. 10C–G; right panels). In conclusion, the results from these simulations suggest that if whole‐cell recording directly from lobular appendages is attempted, judicial choice of recording targets is required to detect exocytosis‐evoked *ΔC*_m_ with high accuracy, with preference for relatively isolated varicosities located further from the soma or apical dendrite (e.g. section #318; Fig. 10B and E).

### Frequency‐dependent activation of voltage‐gated Ca^2+^ currents in AII amacrine cells

**Figure 11:**
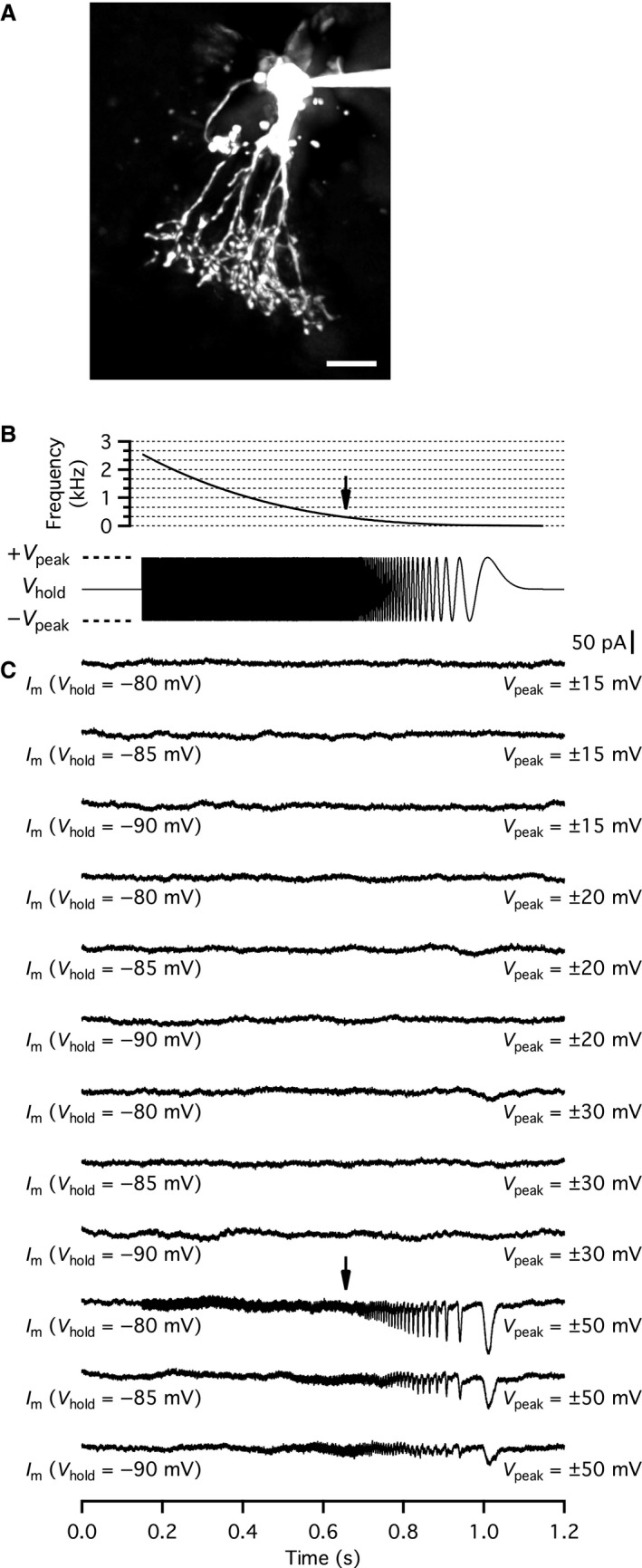
Voltage‐gated currents activated by ZAP stimuli applied in soma recordings of AII amacrine cells. (A) AII amacrine cell filled with Alexa 594 via patch pipette during whole‐cell recording. MIP (along Z axis) generated from wide‐field fluorescence image stack after deconvolution*. *(B) ZAP stimulus (lower trace) with amplitude ± *V*_peak_ from *V*_hold_. The instantaneous frequency of the ZAP stimulus (calculated as the inverse of the intervals defined by the *V*_hold_‐crossing time points) indicated by function in upper panel. It ranged from ~2.5 kHz at the start to ~5 Hz at the end. Time scale in (C) applies to all traces in (B) and (C). (C) Currents evoked by ZAP stimulus in (B) at different values of *V*_hold_ (−80, −85, and −90 mV) and *V*_peak_ (15, 20, 30, and 50 mV). Leak and capacitive currents have been subtracted by a P/N protocol (see Methods). Note the increasing activation of voltage‐gated current with each depolarizing stimulus cycle when the frequency of the ZAP stimulus dropped below ~310 Hz (marked by arrow here and in B) for some combinations of *V*_hold_ and *V*_peak_ (e.g. *V*_hold_ = −80 mV and *V*_peak_ = ±50 mV). Scale bar: 10 *µ*m (A).

In physiological experiments, the most effective way to increase the signal‐to‐noise ratio of “Sine + DC” lock‐in measurements of exocytosis‐evoked *ΔC*_m_ is to increase the peak amplitude of the sine wave voltage‐clamp stimulus (Lindau and Neher 1988; Gillis 1995). It is important, however, that the sine wave does not activate voltage‐gated currents. AII amacrine cells express L‐type Ca^2+^ channels which activate at membrane potentials more positive than −60 mV (when tested with voltage steps) and the primary subcellular location of these channels corresponds to the lobular appendages (Habermann et al. 2003; Balakrishnan et al. 2015). Whether or not a sine wave voltage‐clamp stimulus will activate these Ca^2+^ channels depends on the holding potential, as well as the peak amplitude and the frequency of the sine wave stimulus. To examine the conditions for activation of voltage‐gated Ca^2+^ channels during physiological “Sine + DC” lock‐in measurements with somatic whole‐cell recordings of AII amacrine cells (Fig. 11A), we considered three different holding potentials (*V*_hold_ = −80, −85, and −90 mV) and four different sine wave amplitudes relative to *V*_hold_ (*V*_peak_ = ±15, ±20, ±30, and ±50 mV). To examine how *V*_hold_, *V*_peak_ and sine wave frequency interacted to determine the activation of the voltage‐gated Ca^2+^ current, we employed a 1 sec long ZAP stimulus where the instantaneous frequency was ramped from an initial maximum of ~2.5 kHz to a final minimum of ~5 Hz (Fig. 11B). To subtract linear leak and capacitative currents from the current responses, we applied a scaled‐down version of the ZAP stimulus (see Methods). To isolate Ca^2+^ currents, we used a Cs^+^‐based intracellular solution (to block K^+^ channels) and added TTX to the bath solution (to block voltage‐gated Na^+^ channels). Ligand‐gated channels were blocked by adding CNQX, bicuculline, strychnine, and CPP (see Methods).

Neither a ±15 or a ±20 mV (*V*_peak_) ZAP stimulus applied at *V*_hold_ = −80, −85 or −90 mV activated an inward current (Fig. 11C). With *V*_peak_ = ±30 mV and *V*_hold_ = −80 mV, the ZAP stimulus activated an inward current at the very end of the stimulus period, when the frequency fell below ~20 Hz (4/4 cells; Fig. 11C). When the same stimulus was applied at *V*_hold_ = −85 or −90 mV, no inward current was activated (4/4 cells; Fig. 11C). When *V*_peak_ was increased to ±50 mV, an inward current was activated in phase with the ZAP stimulus when the frequency dropped below ~310 Hz, at all three values of *V*_hold_ (Fig. 11C). The amplitude of the in‐phase current increased gradually with decreasing frequency of the ZAP stimulus (Fig. 11C). In addition to the in‐phase current, we sometimes observed increased current noise during the period with higher frequency of the ZAP stimulus (Fig. 11C), potentially related to imperfect subtraction of leak and capacitive currents. Alternatively, it could correspond to weak activation of Ca^2+^ channels in some lobular appendages.

**Figure 12:**
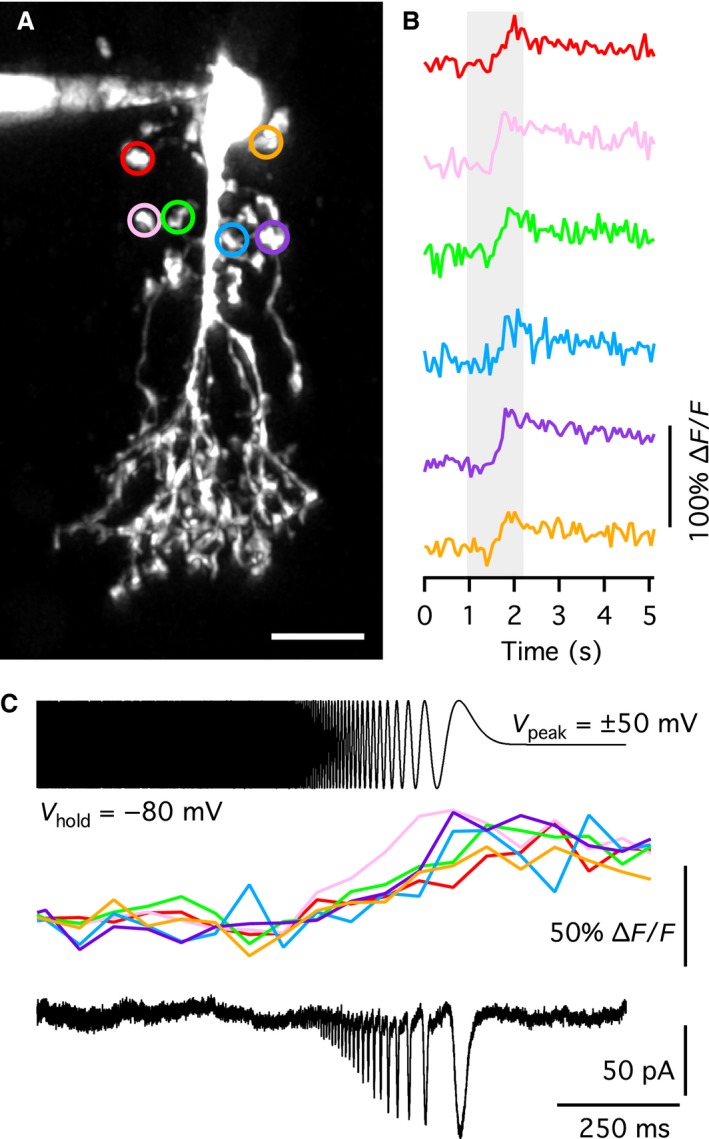
Intracellular Ca^2+^ responses in lobular appendages activated by ZAP stimuli applied to AII amacrine cells. (A) AII amacrine cell filled with Alexa 594 and the Ca^2+^ indicator dye Oregon Green 488 BAPTA‐1 (OGB‐1) via patch pipette during whole‐cell recording. MIP (along *Z* axis) generated from MPE fluorescence image stack after deconvolution. Colored circles correspond to regions of interest (ROIs) over which OGB‐1 fluorescence was measured. (B) Ca^2+^ signals (calculated from OGB‐1 fluorescence) in response to application of ZAP stimulus for each of the ROIs in (A) (indicated by corresponding colors). Gray bar indicates duration of ZAP stimulus with amplitude ± *V*_peak_ = 50 mV from *V*_hold_ = −80 mV (as in Fig. 11B). Each trace is the average of four repetitions. (C) ZAP stimulus (top), Ca^2+^ responses (middle; colored traces as in (B), for the six ROIs indicated in (A)) and evoked whole‐cell currents (bottom; average of three repetitions) displayed at the same time scale. Leak and capacitive currents have been subtracted by a P/N protocol (bottom trace; see Methods). Note increasing activation of voltage‐gated current with each depolarizing stimulus cycle for the lower frequencies of the ZAP stimulus and the corresponding gradual increase of the intracellular Ca^2+^ concentration measured at the lobular appendages (corresponding to the ROIs in (A)). Scale bar: 10 *µ*m (A).

To further investigate this, we performed MPE Ca^2+^ imaging of AII amacrine cells filled with OGB‐1 (for Ca^2+^ imaging) and Alexa 594 (for structural imaging) during whole‐cell recording. We acquired frame scans (~14.5 Hz) at a specific focal plane across a region that covered several lobular appendages. Figure 12A shows a maximum intensity projection of an AII amacrine cell investigated in this way, with colored circles marking six different regions of interest (ROIs), each corresponding to a lobular appendage, where we analyzed potential Ca^2+^ responses evoked by the ZAP stimulus (*V*_hold_ = −80 mV, *V*_peak_ = ±50 mV). For all six ROIs, the ZAP stimulus evoked a frequency‐specific, transient increase of the intracellular Ca^2+^ concentration (measured as *ΔF*/*F*; Fig. 12B). Importantly, the gradual increase of the intracellular Ca^2+^ concentration coincided with the gradually increasing amplitude of the transient, inward currents evoked in phase with the ZAP stimulus (Fig. 12C). This could be seen clearly when we compared the time course of the ZAP stimulus (Fig. 12C, top), the intracellular Ca^2+^ responses (Fig. 12C, middle), and the (leak‐subtracted) current response (Fig. 12C, bottom) side‐by‐side. Similar results were seen for two other AII amacrine cells. These results suggested that the frequency‐dependent inward currents, activated with increasing amplitude and in phase with the ZAP stimulus when the instantaneous frequency dropped below ~310 Hz, involved voltage‐gated Ca^2+^ channels located at the lobular appendages. When a ZAP stimulus does not evoke voltage‐gated Ca^2+^ currents, it is unlikely that it evokes an increase of the intracellular Ca^2+^ concentration.

## Discussion

AII amacrine cells are presynaptic to OFF‐cone bipolar cells and OFF‐ganglion cells at glycinergic synapses made by their large lobular appendages located in the distal part of the inner plexiform layer. One approach to study synaptic release from AII amacrine cells involves simultaneous dual recordings from pairs of synaptically connected cells, that is, an AII amacrine cell and an OFF‐ganglion cell or an AII amacrine and an OFF‐cone bipolar cell (Graydon et al. 2018). Measuring presynaptic capacitance is an alternative that avoids the nonlinearities related to postsynaptic receptor saturation and desensitization, can be used to measure endocytosis, and can provide estimates of the magnitude of the total releasable pool of vesicles when a single presynaptic neuron provides input to several postsynaptic partners (as is the case for AII amacrine cells; Strettoi et al. 1992, 1994). Recently, the “Sine + DC” capacitance measurements of exocytosis in mouse AII amacrines performed by Balakrishnan et al. (2015) revealed a series of important functional properties of synaptic release in this important inhibitory interneuron of the mammalian retina. Here, we verified the ability of the “Sine + DC” technique to measure depolarization‐evoked exocytosis in rat AII amacrine cells. In addition, we used computer simulations with compartmental models of AII amacrine cells to provide evidence that “Sine + DC” capacitance measurements can be used to detect exocytosis. An important result, however, is that for sine wave frequencies ≥1 kHz such measurements are likely to significantly underestimate the magnitudes of the total releasable pools of synaptic vesicles.

### Synaptic release of glycine from AII amacrine cells

Balakrishnan et al. (2015) found evidence for two distinct vesicle pools in AII amacrine cells. This was determined by applying depolarizing pulses of different durations, measuring the saturating values for ∆*C*_m_, and dividing by the capacitance of a single synaptic vesicle. Release of the first pool (corresponding to vesicles docked and primed for exocytosis) resulted in a ∆*C*_m_ of ~34 fF or ~750 vesicles. After additional release of the second pool (corresponding to vesicles clustered near the active zones or docked further away), the total ∆*C*_m_ increased to ~65 fF or ~1400 vesicles. The simulations reported here suggest that these numbers underestimate the true sizes of the vesicle pools, as the sine wave frequency used (2 kHz) will be strongly attenuated along the neurites of AII amacrine cells. For lobular appendages close to the soma, the accuracy can be high. However, for lobular appendages further from the soma, the accuracy will be too low and exocytosis occurring here will not contribute adequately to the measurements. On the other hand, it is difficult to exclude a species difference, potentially related to smaller AII amacrine cells in the mouse retina than in the rat retina. Whereas detailed morphometric data are available for rat AII amacrines, we are not aware of a similar data set for mouse AII amacrines.

For more accurate measurements of total vesicle pool sizes (using somatic recordings), we recommend the use of lower sine wave frequencies, ideally as low as 100 Hz, as the accuracy of ∆*C*_m_ measurements increased continuously with decreasing frequency. A low sine wave frequency, however, will unfortunately introduce increased noise in the measurements, and for a given duration of sine wave stimulation, there will be fewer points to include when averaging capacitance measurements. For measuring the prestimulus baseline capacitance, this can easily be compensated for, but the duration over which the poststimulus capacitance can be measured must be kept short to avoid the effects of endocytosis.

### Optimal conditions for capacitance measurements of exocytosis from AII amacrine cells

Standard capacitance measurements with the “Sine + DC” technique, applied to round cells that can be modeled as simple RC‐circuits, typically use sine wave frequencies in the range 1–5 kHz (Gillis 1995). With round cells, there is no electrotonic attenuation along neurites and these frequencies are sufficiently high to measure exocytosis occuring after variable duration membrane depolarizations with high accuracy and temporal resolution. With somatic whole‐cell recording and sine wave frequencies in the kHz range, the electrotonic attenuation along the neurites of AII amacrine cells is sufficiently strong that a change in capacitance will not be measured with high accuracy in all lobular appendages where exocytosis will be evoked. If we instead use a lower sine wave frequency, for example 100 Hz, to measure exocytosis, the simulations indicate that the measurements are very likely to have high accuracy for all lobular appendages of an AII amacrine cell. The drawback, however, is that the measurements will be compromised by a large junctional conductance of the electrical coupling. This was demonstrated by simulating a small network of electrically coupled AII amacrine cells and is unlikely to differ qualitatively if ON‐cone bipolar cells are incorporated into the network. This is consistent with our previous results from physiological experiments where we found clear transmission of sine wave frequencies of 100 Hz between pairs of electrically coupled AII amacrine cells and pairs of electrically coupled AII amacrine and ON‐cone bipolar cells (Veruki and Hartveit 2002a, 2002b). Using dynamic clamp electrophysiology to implement artificial electrical coupling between AII amacrines, we found that for junctional conductances in the range of 200–3000 pS, the cut‐off frequency (*f*_c_; −3 dB) varied between ~10 and ~40 Hz (Veruki et al. 2008). If a depolarizing stimulus evokes exocytosis from AII and ON‐cone bipolar cells electrically coupled to the stimulated cell, an additional complication of using low sine wave frequencies is that increased capacitance of the coupled cells may be added to the total capacitance measured. To avoid such complications, we suggest two different approaches which should allow the use of a low sine wave frequency (for example 100 Hz). One possibility is to perform the capacitance measurements after blocking electrical coupling pharmacologically with MFA, taking into account that it typically takes up to 30 min before the block is complete (Veruki and Hartveit 2009). Another possibility is to perform capacitance measurements in tissue from animals where the gene for connexin 36 (Cx36) has been deleted to abolish gap junction coupling involving AII amacrine and ON‐cone bipolar cells (Deans et al. 2002). It remains to be determined if the expected increase of noise with low sine wave frequencies (Gillis 1995) will outweigh the expected benefit of including all lobular appendages in the measurements.

In principle, the simulations with AII amacrine cells demonstrate that capacitance measurements from these cells suffer from a similar limitation as was observed previously for rod bipolar cells, when capacitance measurements are performed with somatic whole‐cell recordings. For rod bipolar cells, exocytosis of synaptic vesicles occurs at the axon terminal, that is, furthest from the soma. Simulations performed in our laboratory (Oltedal and Hartveit 2010) demonstrated that for cells with short (32 *µ*m, in contrast to an average length of ~75 *µ*m) and relatively thick axons (~1 *µ*m, in contrast to an average diameter of ~0.7 *µ*m), measurements of exocytosis at the axon terminal with soma‐end recordings can achieve high accuracy even with a sine wave frequency of 800 Hz. For a sine wave frequency of 2 kHz, the accuracy of soma‐end recordings is low both for cells with short/thick axons and for cells with long/thin axons (Oltedal and Hartveit 2010). For AII amacrine cells, the lobular appendages are located close to the soma, but not close enough that a sine wave frequency of for example 2 kHz can be used to detect a change in capacitance from all lobular appendages with high accuracy.

The computer simulations with AII compartmental models suggested that in principle, it might be possible to perform measurements of changes in capacitance with high accuracy when whole‐cell recordings are made directly from individual lobular appendages. To electrotonically isolate the lobular appendage from the rest of the dendritic tree of the AII amacrine and obtain measurements with high accuracy, it was necessary to use high sine wave frequencies, typically ≥10 kHz. There are at least two potential problems associated with the use of such high frequencies. First, a very high frequency can by itself give rise to increased noise (Gillis, 1995). Second, for a sine wave voltage stimulus with high frequency, it is necessary to record the evoked current responses at a correspondingly high bandwidth and the signal‐to‐noise ratio may become too low for adequate measurements.

### Practical considerations for capacitance measurements of AII amacrine cells

Taken together, results from our physiological measurements and computer simulations, suggest the following recommendations for experimental measurements of exocytosis‐evoked ∆*C*_m_ with somatic whole‐cell recordings. First, as long as it is not a goal to estimate the magnitude of the different pools of synaptic vesicles, a sine wave frequency of 1–2 kHz is close to optimal. Measurements with this frequency will not reflect exocytosis occurring at lobulars electrotonically further from the soma, but they are unlikely to be influenced by electrical coupling or by changes in the strength of coupling during recording. It is expected that a capacitance increase of the lobular dendrites also will generate an apparent (transient) increase in *G*_s_ (the series conductance between the tip of the pipette and the interior of the cell). Such changes were also observed in physiological recordings, both for mouse (Balakrishnan et al. 2015) and rat AII amacrine cells (this study).

Second, when we examined different stimulus frequencies (using a ZAP stimulus), holding potentials, and sine wave peak amplitudes, the results suggested that for amplitudes ≤±20 mV, holding potentials of −80 mV and more negative are adequate to avoid activation of voltage‐gated Ca^2+^ channels located at the lobular appendages of AII amacrine cells. For amplitudes ≤±30 mV, a holding potential of −90 mV (or more negative) is preferable when frequencies below ~300 Hz will be used. For amplitudes of ±50 mV, voltage‐gated Ca^2+^ channels are activated at frequencies below ~300 Hz even at a holding potential of −90 mV. However, if the frequency is kept above ~ 300 Hz, it should be possible to avoid activation of voltage‐gated Ca^2+^ channels with holding potentials of −80 mV and more negative. Importantly, however, the exact frequency at which voltage‐gated Ca^2+^ channels are activated by a voltage clamp at the soma is likely to depend on the specific morphology of the cell. These recommendations agree well with an activation threshold of −60 mV for the voltage‐gated Ca^2+^ channels in AII amacrines (Habermann et al. 2003; Balakrishnan et al. 2015).

### Capacitance measurements from morphological structures with arbitrary geometry

When standard capacitance techniques are extended from simple RC‐circuits and applied to morphological structures with arbitrary geometry, the accuracy of measurements designed to detect the increase of capacitance evoked by exocytosis cannot be calculated analytically. Instead, it is necessary to carefully validate any given method by compartmental modeling (Hallermann et al. 2003; Kushmerick and von Gersdorff 2003). For AII amacrine cells, the goal is to quantify the capacitance increase evoked by exocytosis of glycine occurring at the synapses located at their lobular appendages. For measurements of exocytosis occurring at all output synapses on the lobular dendrites of an AII amacrine cell, a depolarizing voltage step applied via a whole‐cell pipette located at the soma is likely to evoke a global depolarization of the complete dendritic arbor, albeit with differences in the speed of voltage clamp for different locations. For measurements of exocytosis occurring at a single lobular appendage, it might in principle be possible to perform such measurements by dendritic whole‐cell recording, but to our knowledge, successful attempts at such recordings have not been published. In our work, we simulated capacitance increases at specific lobular appendages of three different compartmental models of AII amacrine cells (Zandt et al. 2018). In every case, we used the “Sine + DC” technique (Lindau and Neher 1988; Gillis 1995) to quantify the change in capacitance after increasing the surface area by a specific magnitude. Comparing the measured change in capacitance with the implemented change allowed us to evaluate the accuracy for each condition. For the cells investigated, the results clearly indicated that a location‐independent high accuracy can only be reached for a low sine wave frequency (~100 Hz). To estimate the magnitude of the readily releasable pool of synaptic vesicles, a high accuracy for all release sites that contribute to exocytosis is required. It is likely, however, that the release kinetics of the readily releasable pool of vesicles can still be adequately characterized with higher sine wave frequencies (1–2 kHz), as long as the locations contributing to exocytosis do not change systematically during the time it takes to obtain the measurements. These considerations constitute a framework that will need to be taken into account when capacitance measurements of exocytosis are attempted in other types of neurons with similar branched morphology.

## Conflict of Interest

The authors have nothing to disclose.
